# Anti-Adipogenic Effects of *N*-Methylatalaphylline in 3T3-L1 Cells Through Modulation of Metabolic and Mitogenic Signaling Pathways

**DOI:** 10.3390/ijms27093914

**Published:** 2026-04-28

**Authors:** Noppawan Woramongkolchai, Chatchai Chaotham, Utid Suriya, Hnin Ei Ei Khine, Pattara Poungcho, Chaiyaboot Ariyachet, Chia-Hung Yen, Chaisak Chansriniyom

**Affiliations:** 1 Pharmaceutical Sciences and Technology Program, Faculty of Pharmaceutical Sciences, Chulalongkorn University, Bangkok 10330, Thailand; noppawanwora@gmail.com; 2 Center of Excellence in Natural Products and Nanoparticles, Chulalongkorn University, Bangkok 10330, Thailand; p.poungcho@gmail.com; 3 Department of Biochemistry and Microbiology, Faculty of Pharmaceutical Sciences, Chulalongkorn University, Bangkok 10330, Thailand; chatchai.c@chula.ac.th (C.C.); hnineieikhine12@gmail.com (H.E.E.K.); 4 Center of Excellence in Preclinical Toxicity and Efficacy Assessment of Medicines and Chemicals, Chulalongkorn University, Bangkok 10330, Thailand; 5 Department of Biochemistry, Faculty of Science, Mahidol University, Bangkok 10400, Thailand; utid.sur@mahidol.ac.th; 6 Department of Biochemistry, Faculty of Medicine, Chulalongkorn University, Bangkok 10330, Thailand; chaiyaboot.a@chula.ac.th; 7 Graduate Institute of Natural Products, College of Pharmacy, Kaohsiung Medical University, Kaohsiung 807, Taiwan; chyen@kmu.edu.tw; 8 Department of Pharmacognosy and Pharmaceutical Botany, Faculty of Pharmaceutical Sciences, Chulalongkorn University, Bangkok 10330, Thailand

**Keywords:** *N*-methylatalaphylline, adipogenesis, PI3K-AKT, MAPK, AMPK-ACC

## Abstract

Adipogenesis is a critical factor in causing obesity, which is a global health problem associated with metabolic disorders, such as insulin resistance and cardiovascular diseases. Natural compounds with anti-adipogenic activity may represent potential approaches for modulating adipocyte function. However, despite increasing interest in natural products, the anti-adipogenic potential of acridone alkaloids, particularly prenylated derivatives, remains largely unexplored. This study examined the effects of *N*-methylatalaphylline (NMA), a prenylated acridone alkaloid, on adipocyte differentiation, lipid accumulation, and glucose uptake. NMA exhibited anti-adipogenesis, particularly toward preadipocytes, and significantly reduced lipid accumulation in murine 3T3-L1 and human PCS-210-010 adipocytes at nontoxic doses (1.5–6 µM). At 3–6 µM, NMA downregulated adipogenic regulators, including PPARγ, C/EBPα, and SREBP1, along with adipogenic effectors, such as FABP4, adiponectin, LPL, PLIN1, and FAS. Mechanistic studies indicated that NMA treatment was associated with reduced phosphorylation of AKT, ERK, and p38, accompanied by cell cycle arrest and inhibition of mitotic clonal expansion. Meanwhile, activation of AMPK-ACC signaling, which may contribute to suppression of adipogenesis and reduced glucose uptake, was observed in differentiated 3T3-L1 cells after treatment with 6 µM NMA for 48 h. Additionally, molecular docking and molecular dynamics simulations suggested potential interaction between NMA and ERK1, supported by hydrogen bonding and hydrophobic contacts. Overall, these findings suggest that NMA exerts anti-adipogenic effects in vitro by modulating adipocyte proliferation, differentiation, and lipid metabolism. These findings highlight NMA as a promising acridone alkaloid scaffold for anti-adiposity applications, warranting further in vivo validation.

## 1. Introduction

Obesity is an emerging health issue worldwide in which one in eight people develops excessive body fat (body mass index (BMI) > 30), which results in a life-threatening condition [1]. Over-adiposity in the abdominal visceral depot and vital organs, such as the heart and liver, is associated with insulin resistance, coronary artery disease, and fatty liver [2]. Increases in the number and size of adipocytes, respectively known as adipocyte hyperplasia and hypertrophy, are influenced by insulin-mediated glucose uptake, *de novo* lipogenesis, and fatty acid uptake [3,4]. Recently, FDA-approved peptide drugs, including semaglutide and tirzepatide, act as glucagon-like peptide 1 (GLP-1) receptor agonists. They enhance adipolysis, reduce food intake, and delay gastric emptying for weight management and anti-obesity [5]. However, limitations such as high cost, side effects, and long-term safety concerns have prompted the search for alternative therapeutic agents. Intriguingly, several studies have highlighted the potential of natural products as alternative anti-obesity therapies because of their inhibitory effects on adipogenesis and lipogenesis by interfering with multiple target pathways involved in adipocyte development and function. For example, epigallocatechin-3-gallate, a flavan-3-ol gallate in green tea, suppressed the expression of adipogenic regulators and adipogenic effectors [peroxisome proliferator-activated receptor γ (PPARγ) and fatty acid synthase (FAS)] by inhibiting the phosphatidylinositol-3-kinase (PI3K)/protein kinase B (PKB, also known as AKT) pathway and decreasing adipocyte proliferation by modulating the extracellular signal-regulated kinase (ERK) and cyclin-dependent kinase 2 (CDK2) pathways [6,7].

Acridone alkaloids are a class of secondary metabolites predominantly distributed in the Rutaceae plant family, and most of them exhibit anticancer and antimalarial activities [8,9]. Recent studies have indicated that the anti-skeletal muscle (C2C12) cell proliferation activity of 1,3-dihydroxyacridones is mediated by the inhibition of the AKT pathway [10]. In addition, Gao et al. [11] reported that a 4-prenylated furanoacridone, buxifoliadine, exhibited antiproliferative activity against hepatoblastoma (HepG2) cells by suppressing the ERK pathway. Moreover, acrifoline was active against several kinases, particularly dual-specificity tyrosine phosphorylation-regulated kinase 1A (DYRK1A), which functions in neuronal development [12]. These findings suggest that acridone alkaloids, particularly prenylated derivatives, may act as modulators of kinase-driven signaling pathways that are also implicated in adipogenesis. Acridone alkaloids have inhibitory effects on protein kinase; however, their roles in metabolic diseases and adipogenesis remain largely unexplored. Adipogenesis includes preadipocyte proliferation (mitotic clonal expansion, MCE), differentiation, and lipid accumulation, and involves kinase-mediated signaling pathways in the insulin-PI3K-AKT, ERK mitogen-activated protein kinase (MAPK), and p38 MAPK pathways during adipocyte differentiation [13,14].

Based on these considerations, prenylated acridone alkaloids were selected in this study due to their structural features and reported kinase-modulating activities, which may be relevant to the regulation of adipocyte proliferation, differentiation, and maturation. In this study, two prenylated acridone alkaloids [*N*-methylatalaphylline (NMA) and *N*-methylcyclo-atalaphylline-A (NMCA)] were subjected to primary screening for cell viability, lipid accumulation, and glucose uptake activity in mouse and human adipocytes. The active compound was evaluated in terms of its molecular mechanisms and in silico prediction.

## 2. Results

### 2.1. Effects of NMA and NMCA on Cell Viability and Cellular Lipid Content in 3T3-L1 Cells

**Figure 1:**
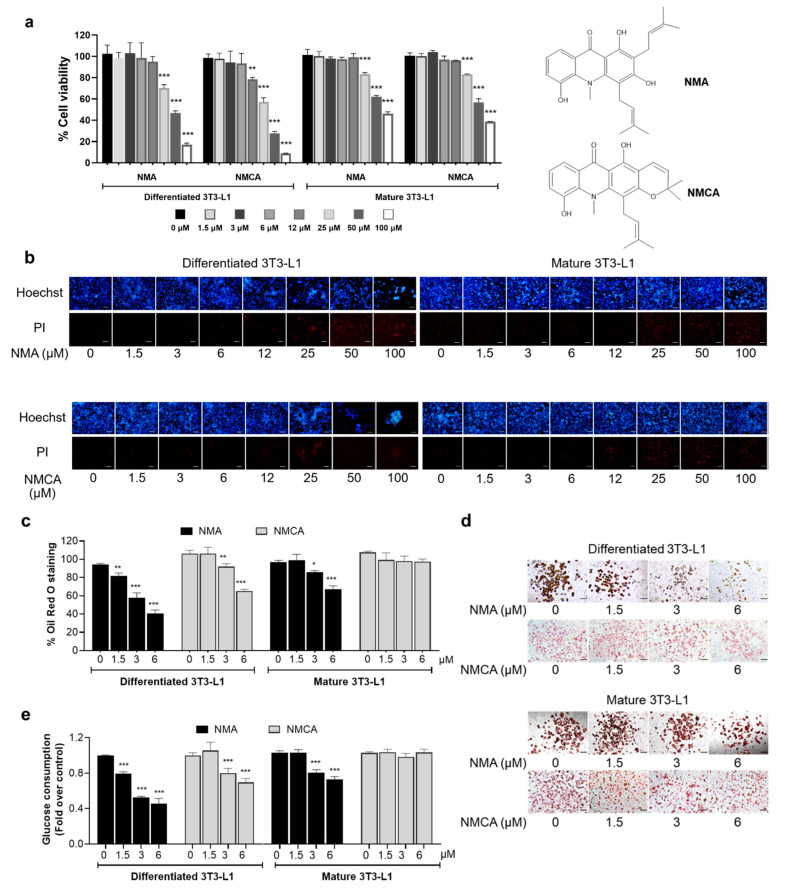
Effect of NMA and NMCA on cell viability, cellular lipid metabolism, and glucose uptake. (**a**) The MTT viability assay was performed on differentiated, mature 3T3-L1 cells after treatment with NMA and NMCA over 1.5–100 μM for 48 h. (**b**) Cells stained with Hoechst 33342 and PI were observed under a fluorescence microscope (scale bar  =  100 μm). (**c**) The percentage and (**d**) representative images (scale bar  =  100 μm) of existing ORO staining for lipid accumulation assay, and (**e**) relative glucose consumption were measured in 3T3-L1 cells after treatment with nontoxic concentrations of NMA and NMCA (1.5–6 μM). The error bars represent ± SD of triplicate data. * *p* < 0.05, ** *p* < 0.01, *** *p* < 0.001 show statistical significance compared to the control (without compounds).

NMA and NMCA (1.5–100 μM) were evaluated for their cytotoxicity in murine 3T3-L1 cells at differentiating and mature stages using the MTT assay. Both compounds exhibited higher cytotoxicity toward differentiated versus mature adipocytes, particularly NMCA [half-maximal cytotoxicity concentration (CC_50_): 31.94 and 67.04 μM for differentiated and mature adipocytes, respectively], whereas NMA showed less cytotoxicity (CC_50_: 45.90 and 81.73 μM for differentiated and mature adipocytes, respectively) (Figure 1a). The results indicated that 1.5–6 μM NMA and NMCA were nontoxic to the cells. In addition, nuclear staining showed consistent results. Neither bright blue Hoechst 33342 nor red propidium iodide (PI) fluorescence was observed at 1.5–6 μM for both compounds, suggesting that neither apoptosis nor necrosis occurred (Figure 1b).

Nontoxic doses of NMA (1.5–6 μM) inhibited lipid accumulation in 3T3-L1 adipocytes in a dose-dependent manner, with significant effects observed at 3 and 6 μM (*p* < 0.001) (Figure 1c,d). NMA reduced Oil Red O (ORO) staining by 56.6% at 6 μM in differentiated cells, with a half-maximal effective concentration (EC_50_) of 4.43 μM—approximately one-tenth of its CC_50_—and by 30.7% in mature adipocytes. In comparison, NMCA reduced ORO staining by 13.5% and 38.9% at 3 and 6 μM, respectively, in differentiated cells, but showed no effect in mature adipocytes at the highest nontoxic dose (6 μM).

### 2.2. Effects of NMA and NMCA on Glucose Uptake in 3T3-L1 Cells

As glucose is a fuel, it is broken down and turned into fatty acids by *de novo* lipogenesis in adipocytes. Therefore, the effects of NMA and NMCA on glucose uptake in 3T3-L1 cells in the differentiating and mature phases were examined. Both compounds exhibited a dose-dependent inhibitory effect on differentiated 3T3-L1 adipocytes, particularly NMA, which significantly reduced glucose uptake by 21% at the lowest dose (1.5 μM, *p* < 0.001) (Figure 1e). In contrast, in mature 3T3-L1 cells, NMA markedly reduced glucose uptake by 22% and 29% at 3 and 6 μM (*p* < 0.001), respectively, whereas NMCA did not inhibit glucose uptake at the maximum dose of 6 μM in mature 3T3-L1 cells. NMA was chosen for further study because it showed more pronounced effects on lipid droplet reduction and glucose consumption than NMCA, especially in differentiated adipocytes.

### 2.3. Effect of NMA on Glucose Transporter (GLUT) Expression in 3T3-L1 Cells

**Figure 2:**
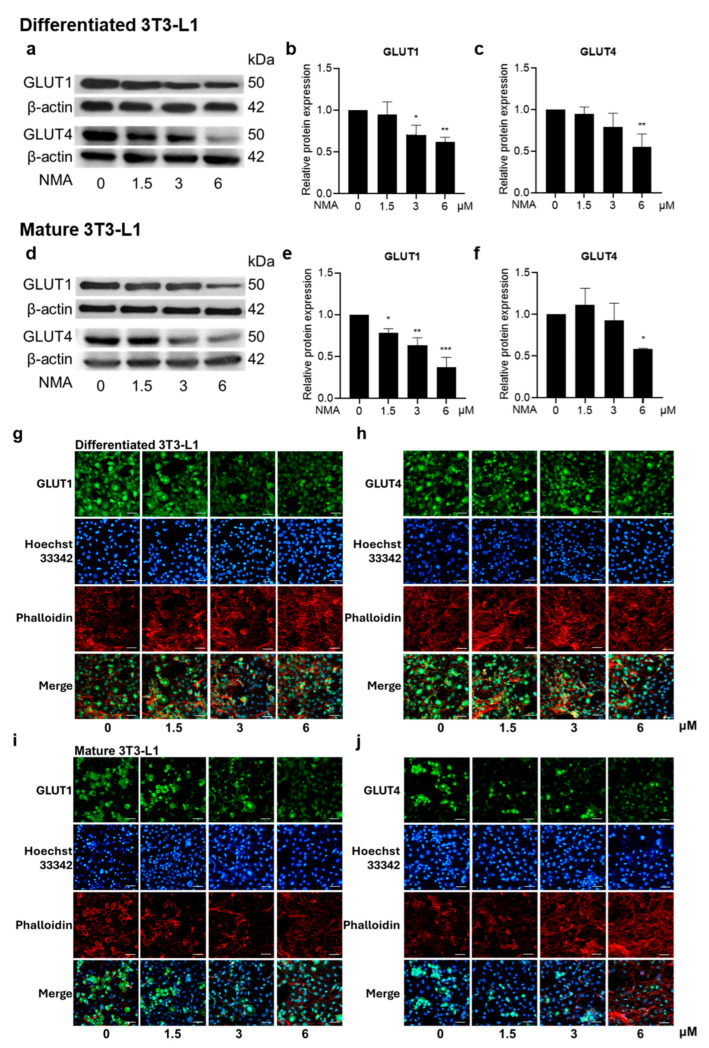
Effect of NMA on the expression of GLUTs in 3T3-L1 cells. Cells were treated with NMA (1.5–6 μM) for 48 h. Western blotting images demonstrated the expressions of GLUT1 and GLUT4 in (**a**) differentiated and (**d**) mature 3T3-L1. The relative protein expressions of GLUT1 and GLUT4 were determined using ImageJ software version 1.53r (National Institutes of Health, Bethesda, MD, USA) and normalized against β-actin level (differentiated cells: (**b**) GLUT1 and (**c**) GLUT4, mature cells: (**e**) GLUT1 and (**f**) GLUT4). The error bars represent ± SD of triplicate data. * *p* < 0.05, ** *p <* 0.01, *** *p <* 0.001 show statistical significance compared to the control (without NMA). Immunofluorescence represented GLUT1 and GLUT4 on differentiated 3T3-L1 ((**g**) GLUT1; (**h**) GLUT4) and mature 3T3-L1 ((**i**) GLUT1; (**j**) GLUT4). The GLUT1 and GLUT4 were labeled with green fluorescence from specific secondary antibodies. The nucleus of the cells was labeled in blue using Hoechst 33342. Actin staining with phalloidin was shown in red pseudocolor. The merged picture illustrated the locations of the nucleus, GLUT, and cytoskeleton. The images were magnified at ×20 under a confocal microscope (scale bar  =  50 μm).

In differentiated and mature 3T3-L1 cells, the effect of NMA on GLUT1 and GLUT4 protein expression was determined by Western blot analysis (Figure 2a,d). NMA significantly reduced GLUT1 and GLUT4 expression in both differentiating and mature 3T3-L1 cells in a concentration-dependent manner. In differentiating adipocytes, GLUT1 expression significantly decreased to 0.70 and 0.62 at 3 and 6 μM, respectively (approximately 30–38% reduction, Figure 2b), while GLUT4 expression decreased to 0.79 and 0.55 (approximately 21–45% reduction, Figure 2c). These reductions indicate a strong inhibitory effect of NMA on glucose transporter expression during the differentiation stage. In mature adipocytes, NMA also reduced GLUT expression, although the pattern was less active. GLUT1 expression decreased to 0.63 and 0.38 at 3 and 6 μM, respectively (approximately 37–62% reduction, Figure 2e). GLUT4 expression showed minimal change at lower concentrations (0.93 at 3 μM) but decreased to 0.58 at 6 μM (approximately 42% reduction, Figure 2f). Overall, the inhibitory effects of NMA on GLUT1 and GLUT4 expression were stronger during the differentiation stage, whereas in mature adipocytes, significant reductions were primarily observed at higher concentrations.

In addition, the decrease in GLUT expression induced by NMA was observed using immunofluorescence staining. NMA at 1.5–6 μM decreased GLUT1 and GLUT4 expressions in both the differentiating and mature stages of 3T3-L1 cells (Figure 2g–j).

### 2.4. Effect of NMA on MCE

**Figure 3:**
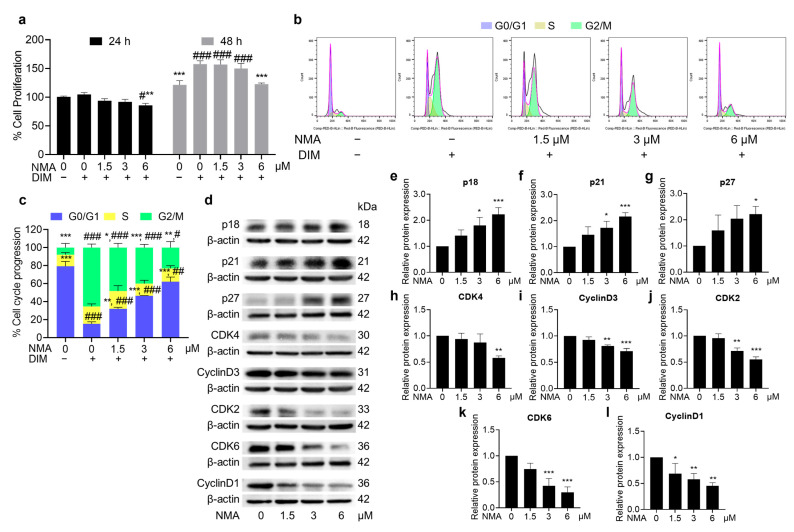
Effect of NMA on cell proliferation and cell cycle progression in differentiated 3T3-L1 cells. Cells were cultured either with or without DIM, and those cultured with DIM were additionally incubated with NMA (1.5–6 μM) for 24 and 48 h. (**a**) The percentage of cell proliferation using crystal violet assay. (**b**) Cell cycle analysis was performed using flow cytometry after the cells were exposed to NMA for 48 h. (**c**) Cell population in G0/G1, S, and G2/M phases in the cell cycle was presented in the percentage. (**d**) Western blotting image represented the expression of some MCE-related proteins [p18, p21, p27, CDK4, Cyclin D3, CDK2, CDK6, and cyclin D1] in the NMA-treated cells, and (**e**–**l**) their relative protein expressions were normalized against β-actin level and measured using ImageJ version 1.53r. The error bars represent ± SD of triplicate data. * *p* < 0.05, ** *p* < 0.01, *** *p* < 0.001 show statistical significance compared to the control with DIM at the same time. # *p* < 0.05, ## *p <* 0.01, ### *p <* 0.001 show statistical significance compared to the control without DIM at the same time.

NMA inhibited 3T3-L1 preadipocyte proliferation in a crystal violet assay, particularly at 6 μM. NMA-treated cells showed a significant decrease in proliferation at 24 and 48 h in the presence of differentiation media (DIM) (Figure 3a). This effect also occurred in a dose-dependent manner, increasing the cell population in the G0/G1 phase and decreasing the cell population in the S and G2/M phases of the cell cycle, as determined by flow cytometry (Figure 3b). In 6 μM NMA-treated cells, approximately 62% of preadipocytes remained in a quiescent state (G0/G1 phase) compared with those in NMA-untreated cells (15% of preadipocytes), indicating an effect on cell cycle arrest (Figure 3c). The expression of MCE proteins (cyclin D1, cyclin D3, CDK2, CDK4, and CDK6), which affect cell cycle progression, decreased, whereas the expression of cell cycle suppressors (p18, p21, and p27) increased (Figure 3d–l), particularly in the 6 μM NMA treatment.

### 2.5. Effect of NMA on Adipogenic Regulators and Adipogenic Effectors

**Figure 4:**
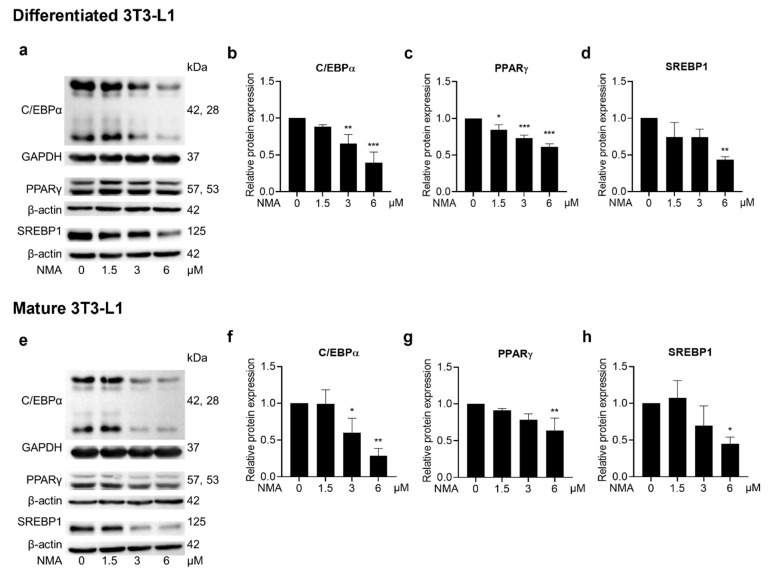
Effect of NMA on the expression of key adipogenic regulators. Cells were treated with NMA (1.5–6 μM) for 48 h. Western blotting images showed the expression of C/EBPα, PPARγ, and SREBP1 in (**a**) differentiated and (**e**) mature 3T3-L1. The relative protein expressions of C/EBPα, PPARγ, and SREBP1, normalized against β-actin or GAPDH level, were measured using ImageJ version 1.53r. (differentiated cells: (**b**) C/EBPα, (**c**) PPARγ, and (**d**) SREBP1, mature cells: (**f**) C/EBPα, (**g**) PPARγ, and (**h**) SREBP1). The error bars represent ± SD of triplicate data. * *p* < 0.05, ** *p <* 0.01, *** *p <* 0.001 show statistical significance compared to the control (without NMA).

**Figure 5:**
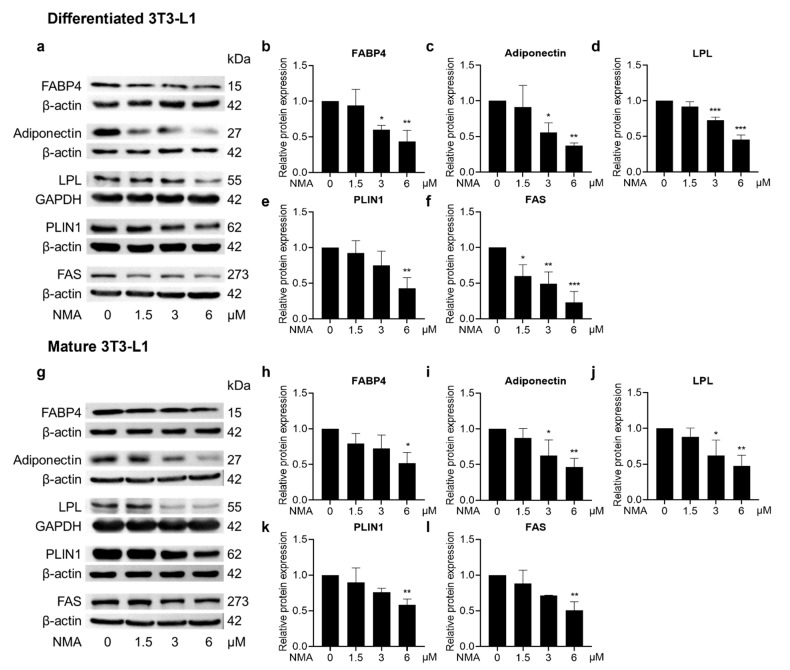
Effect of NMA on the expression of adipogenic effectors. Cells were treated with NMA (1.5–6 μM) for 48 h. Western blotting images showed the expression of FABP4, adiponectin, LPL, PLIN1, and FAS in (**a**) differentiated and (**g**) mature 3T3-L1. The relative protein expressions of FABP4, Adiponectin, LPL, PLIN1, and FAS, normalized against β-actin or GAPDH level, were measured using ImageJ version 1.53r. (differentiated cells: (**b**) FABP4, (**c**) Adiponectin, (**d**) LPL, (**e**) PLIN1, (**f**) FAS, mature cells: (**h**) FABP4, (**i**) Adiponectin, (**j**) LPL, (**k**) PLIN1, (**l**) FAS). The error bars represent ± SD of triplicate data. * *p* < 0.05, ** *p <* 0.01, *** *p <* 0.001 show statistical significance compared to the control (without NMA).

A mechanistic study of NMA in adipogenesis was conducted. Western blot analysis was used to quantify the expression of adipogenesis-related proteins in 3T3-L1 cells. A marked decrease in CCAAT/enhancer-binding protein α (C/EBPα), PPARγ, and sterol regulatory element-binding protein 1 (SREBP1) protein expression was observed in 3–6 μM and 6 μM NMA-treated differentiated and mature 3T3-L1 cells, respectively (Figure 4a–h). Moreover, expression of adipogenic effector proteins [fatty acid-binding protein 4 (FABP4), adiponectin, lipoprotein lipase (LPL), perilipin 1 (PLIN1), and FAS] was reduced in the 3 and 6 μM NMA-treated differentiated 3T3-L1 cells (Figure 5a–f). In contrast, the inhibitory effect of NMA on adipogenic effector protein expression in mature 3T3-L1 was less effective compared with that in the differentiating stage, which required 6 μM NMA to cause a significant reduction (Figure 5g–l).

### 2.6. Effect of NMA on Phosphorylated Proteins Involved in Adipocyte Differentiation-Related Metabolic and Mitogenic Signaling Pathways

**Figure 6:**
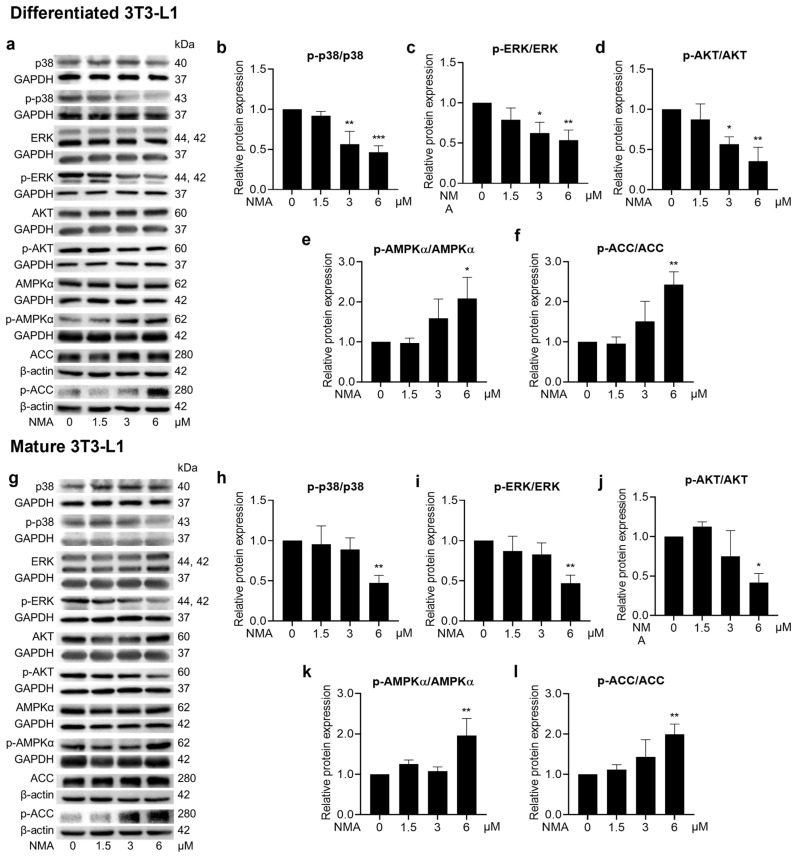
Effect of NMA on the phosphorylation of regulated proteins in adipogenic pathways. Cells were treated with NMA (1.5–6 μM) for 48 h. Western blotting images depicted the expression of phosphorylated (with p prefix) and unphosphorylated proteins of p38, ERK, AKT, AMPKα, ACC in (**a**) differentiated and (**g**) mature 3T3-L1. The protein expressions demonstrated as the ratio of phosphorylated to unphosphorylated forms of p38, ERK, AKT, AMPKα, ACC, normalized against β-actin or GAPDH level, were measured using ImageJ version 1.53r. (differentiated cells: (**b**) p-p38/p38, (**c**) p-ERK/ERK, (**d**) p-AKT/AKT, (**e**) p-AMPKα/AMPKα, (**f**) p-ACC/ACC, mature cells: (**h**) p-p38/p38, (**i**) p-ERK/ERK, (**j**) p-AKT/AKT, (**k**) p-AMPKα/AMPKα, (**l**) p-ACC/ACC). The error bars represent ± SD of triplicate data. * *p* < 0.05, ** *p <* 0.01, *** *p <* 0.001 show statistical significance compared to the control (without NMA).

NMA at 3 and 6 μM exhibited significant reductions in p-p38/p38, p-ERK/ERK, and p-AKT/AKT in differentiated 3T3-L1 cells (Figure 6a–d). Meanwhile, a lesser effect of NMA was observed in mature 3T3-L1 cells at 6 μM (Figure 6g–j). The decrease in phosphorylation of adipogenic kinases was consistent with a reduction in lipid accumulation as determined by the ORO staining assay (Figure 1c,d). The strong suppressive effect of NMA on these kinases during early differentiation, compared with the mature stage, suggests that its primary mechanism of action is more pronounced in inhibiting cell proliferation, MCE, and glucose uptake-related pathways. Regarding the reduced glucose uptake activity of NMA, a reduction in phosphorylated AKT highlights this phenomenon. NMA at 6 μM most prominently decreased the p-AKT/AKT ratio (Figure 6d), corresponding to the reduction in glucose uptake observed during early differentiation. In addition, the significant change in the phosphorylation ratio of p-AKT/AKT in mature adipocytes was noted only at a dose of 6 μM (Figure 6j). The increased p-AMPKα/AMPKα and p-ACC/ACC ratios in NMA-treated cells (Figure 6e,f,k,l) suggested activation of the AMP-activated protein kinase (AMPK)–acetyl-CoA carboxylase (ACC) cascade, which inhibits fatty acid production via *de novo* lipogenesis and decreases the levels of lipogenic effector proteins, such as FAS and FABP4.

### 2.7. Effects of NMA on Cell Viability, Lipid Accumulation, and Glucose Uptake in PCS-210-010 Cells

**Figure 7:**
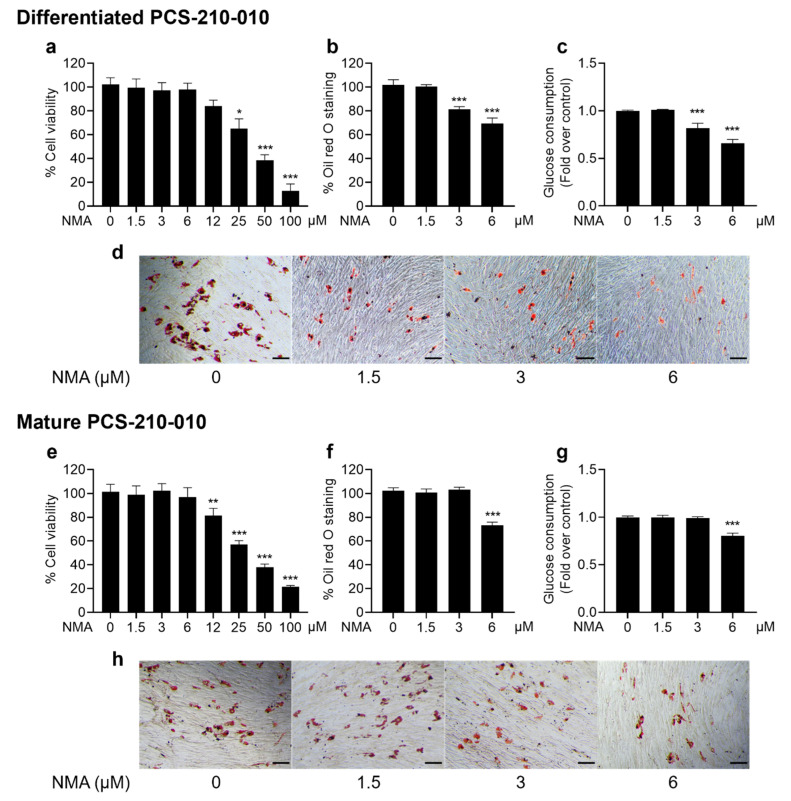
Effects of NMA on cell viability, lipid accumulation, and glucose uptake in PCS-210-010 cells. Cells were incubated with NMA for 48 h (1.5–100 μM) for MTT assay. ((**a**) differentiated, (**e**) mature), whereas the non-cytotoxic concentration of NMA (1.5–6 μM) was treated in the cells to determine the percentage of ORO staining ((**b**) differentiated, (**f**) mature), and relative glucose consumption ((**c**) differentiated, (**g**) mature). The error bars represent ± SD of triplicate data. * *p* < 0.05, ** *p <* 0.01, *** *p <* 0.001 show statistical significance compared to the control (without NMA). Representative images of ORO staining for lipid droplets ((**d**) differentiated, (**h**) mature), scale bar  =  100 μm.

We demonstrated the anti-adipogenic effect of NMA in primary human PCS-210-010 differentiated and mature adipocytes. The results indicated that NMA at concentrations of 1.5–6 μM was safe for human PCS-210-010-differentiated and mature adipocytes (Figure 7a,e). Lipid accumulation was significantly decreased in the 3 and 6 μM, and 6 μM NMA-treated groups for differentiated and mature adipocytes, respectively (Figure 7b,f), which was consistent with a reduction in glucose consumption (Figure 7c,g).

### 2.8. Protein Kinases’ Binding Affinity: Molecular Docking

**Figure 8:**
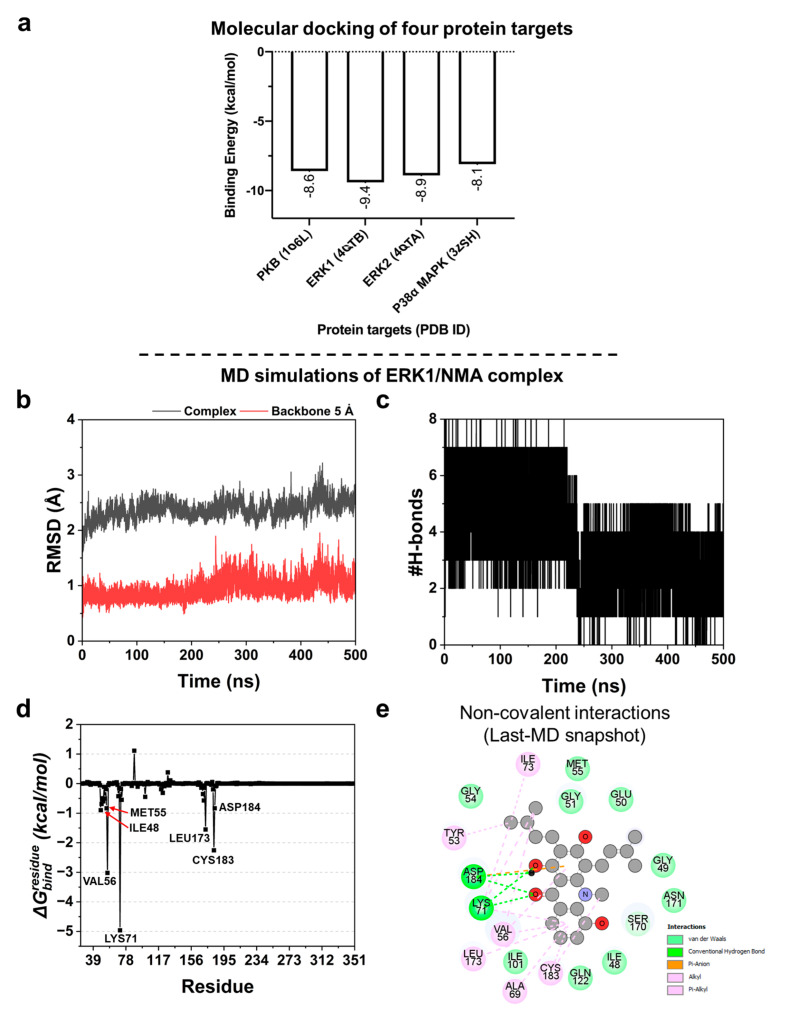
Protein-binding affinity analysis by Autodock VinaXB program. (**a**) Predicted binding energy (kcal/mol) of protein targets with NMA. Structural dynamics and ligand recognition of ERK1/NMA complex by MD simulations. (**b**) Analysis of the whole complex and backbone RMSD of amino acids within the 5 Å around the ligand. (**c**) Numbers of H-bond formation throughout the 500 ns. (**d**) Free energy decomposition to each amino acid (${\Delta G}_{bind}^{residue}$) calculated by using the MM/GBSA method. (**e**) 2D non-covalent interactions based on the last MD snapshot, visualized by the BIOVIA Discovery Studio Visualizer 2021, with labels of different colors denoting different interactions.

Based on Western blot analysis (Figure 6b–d), NMA reduced the expression of several cellular proteins, resulting in decreased lipid accumulation. To further explore potential molecular targets, molecular docking was performed to predict protein–ligand binding affinities and evaluate interactions with four key signaling proteins: PKB (AKT), ERK1, ERK2, and p38α MAPK. As shown in Figure 8a, NMA exhibited binding to all four targets, with comparable binding energies ranging from −9.4 to −8.1 kcal/mol, suggesting potential interactions with multiple signaling proteins. Among these, slightly stronger binding affinities were observed for ERK1 and ERK2 (−9.4 and −8.9 kcal/mol, respectively).

Given the marginally lower binding energy, ERK1 was selected for further molecular dynamics (MD) simulations to investigate protein–ligand stability at the atomic level. However, the relatively small differences in binding energies across targets suggest that NMA may act not through a single dominant target but through multi-target interactions. This is consistent with the modulation of multiple signaling pathways observed in the Western blot analysis.

### 2.9. MD Simulations of ERK1/NMA Complex

MD simulations were conducted to gain insight into the formation of the NMA-ERK1 complex in near-physiological and dynamic surroundings. To assess binding stability, the whole complex root-mean-square deviation (RMSD) and backbone RMSD of the amino acids within 5 Å around the ligand were calculated. As shown in Figure 8b, the RMSD values exhibited a slight deviation, particularly at the first 50 ns (seen in a black line for complex RMSD) and around 430–450 ns for both the complex and backbone 5 Å RMSD values (Figure 8b). Subsequently, they became stable throughout the last 50 ns (450–500 ns), suggesting that the protein-ligand complex may reach equilibrium and form a stable complex with human ERK1. Thus, the MD snapshots at 450–500 ns were used for further analyses. Furthermore, the number of hydrogen bonds (H-bond) was estimated throughout the simulation. We found that NMA could have up to eight H-bonds and become stable at four bonds after 250 ns (Figure 8c). The amino acid residues capable of forming H-bonds with NMA include Lys71 and Asp184, as visualized by the last MD snapshot (Figure 8e). In addition, the free energy decomposition (${\Delta G}_{bind}^{residue}$) was measured using the MM/GBSA method to identify the hot-spot residues for ligand binding, considering only the residues with ${\Delta G}_{bind}^{residue}$ values less than −0.80 kcal/mol. We identified seven key binding amino acids, including Ile48, Met55, Val56, Lys71, Leu173, Cys183, and Asp184 (Figure 8d), which play an important role in NMA binding. The non-covalent interactions based on the last MD snapshot revealed that complex formation was predominantly driven by hydrophobic interactions (alkyl and pi-alkyl) followed by van der Waals (vdW) forces, H-bonds, and electrostatic interaction, respectively (Figure 8e).

## 3. Discussion

Acridone alkaloids have attracted attention due to their ability to modulate key signaling pathways, such as PI3K-AKT and ERK, which are critically involved in adipocyte differentiation and fat cell development [10,11]. In this research, acridone alkaloids, NMA and NMCA, at a nontoxic concentration range (1.5–6 μM) were evaluated for their inhibition activity on lipid accumulation in 3T3-L1 and PCS-210-010 cells. Previously, NMA and NMCA exhibited cytotoxicity against prostate cancer (LNCaP) cells, with NMA showing 33.07% cell viability at 100 μM, a cytotoxic effect comparable to that of NMCA [11]. In addition, NMA exhibited lower cytotoxicity than NMCA against human cholangiocarcinoma (HuCCA-1), human lung cancer (A549), HepG2, and acute lymphoblastic leukemia (MOLT-3) cell lines [15]. NMA is an acridone alkaloid found in many Rutaceous plants, such as *Atalantia buxifolia*, *A. monophylla*, *Glycosmis parva*, *G. trichanthera*, *and G. ovoidea*, whereas NMCA from *A. monophylla* and *G. parva* has been reported [9]. To our knowledge, this study is the first to report the anti-adipogenic activity of acridone alkaloids under in vitro conditions. The reduction in ORO staining by NMA and NMCA indicated decreased cellular lipid accumulation in 3T3-L1 cells, particularly during the early differentiation phase. Moreover, NMA exhibited a stronger inhibitory effect on lipid droplet accumulation than NMCA, with lower cytotoxicity, and was therefore selected for further mechanistic investigation.

Given that lipid accumulation during adipocyte differentiation depends on glucose availability as a substrate for lipogenesis, we further investigated the compounds’ effects on glucose uptake. Reduced glucose uptake may limit the availability of substrates for lipid synthesis [3]. The relationship between the percent reductions in lipid droplet accumulation and glucose uptake was strongly correlated in differentiated adipocytes treated with NMA (*r* = 0.956), followed by mature adipocytes treated with NMA (*r* = 0.841) (Figure 1c,e). In contrast, a weaker correlation coefficient (*r* = 0.687) was observed in NMCA-treated differentiated adipocytes, suggesting a potential link between both processes. Additionally, the decrease in GLUT4 and GLUT1 expressions (Figure 2a–j) observed in NMA-treated 3T3-L1 cells may be associated with reduced glucose consumption, thereby limiting lipid accumulation. However, the causal relationship between glucose metabolism and the anti-adipogenic effect requires further investigation.

Because NMCA showed greater cytotoxicity and weaker inhibitory effects on lipid droplet accumulation and glucose uptake than NMA, NMA was selected for further studies of the molecular mechanisms underlying anti-adipogenesis. During adipocyte differentiation, preadipocyte proliferation is an early event in adipocyte hyperplasia; the cells undergo the MCE process, thereby increasing cell numbers, during which the levels of preadipocyte cyclin/CDK complexes increase, while the cell cycle suppressors decrease [16]. In NMA-treated cells, progression in the MCE phase was suppressed, resulting in a large population of growth-arrested preadipocytes remaining in the G0/G1 phase. Decreased cyclin/CDK protein expression and increased protein expression of negative regulators were observed in the corresponding phases of the cell cycle [G1 phase: ↓cyclin D1/D3, ↓CDK4/6, ↑p18; G1/S phase ↓CDK2, ↑p21, ↑p27; and S phase: ↓CDK2, ↑p21], which indicates the suppression of cell proliferation (Figure 3).

This suppression of cell proliferation prompted us to assess whether NMA also altered adipogenic transcription factors and their downstream targets (Figure 4 and Figure 5). In the present study, NMA treatment significantly reduced the protein expression of C/EBPα, PPARγ, and SREBP1, particularly during early differentiation, suggesting inhibition of adipocyte differentiation. These transcription factors play key roles in adipogenesis through a coordinated transcriptional cascade. The expression of these proteins was phase-dependent, with the duration of growth and adipocyte development influencing their levels. C/EBPα and PPARγ control terminal differentiation and lipid metabolism, while SREBP1 promotes lipogenic gene expression [13,16,17]. Consistent with this, NMA treatment also reduced the expression of downstream adipogenic effectors, including FAS, PLIN1, LPL, adiponectin, and FABP4, which are involved in lipid storage, fatty acid uptake, and adipocyte maturation [17,18,19,20,21]. This coordinated downregulation of adipogenic transcription factors and their target genes may contribute to the reduced lipid accumulation observed in NMA-treated cells.

Because adipocyte differentiation is governed by multiple signaling cascades, we next investigated whether the anti-adipogenic effects of NMA were associated with modulation of PI3K-AKT and MAPK pathways (Figure 6). In this study, NMA treatment significantly decreased the phosphorylation levels of AKT, ERK, and p38 MAPK, particularly during the early differentiation stage, which was consistent with the observed reduction in lipid accumulation. The PI3K-AKT pathway is directly responsible for the effect of insulin, whereas the ERK/MAPK pathway is also activated by insulin through the Ras-MEK cascade [22,23]. MCE is an essential process in early adipogenesis, partly regulated by the ERK/MAPK signaling pathway. Inhibition of MEK1 by PD98059 or U0126 attenuated the expression of PPARγ and C/EBPα, decreased the cyclin A-CDK2 complex, and suppressed adipogenic marker formation (FABP4 and PLIN 1) [24,25]. ERK controls cyclin D1 transcription and facilitates cyclin–CDK complex formation during the G1/S phase [26]. The PI3K-AKT pathway also cooperates with the ERK/MAPK pathway to promote cell proliferation and cell cycle entry by regulating cyclins and cell cycle inhibitors [26]. The phosphorylation of AKT at Ser-473 promotes 3T3-L1 differentiation, increases the nuclear fraction of PPARγ, and results in lipid accumulation [27]. In addition, AKT activation regulates SREBP1c transcription via mechanistic target of rapamycin complex1 (mTORC1), enhances FAS activity, and promotes lipid production [28]. p38 MAPK is also one of the kinases whose phosphorylation is observed during the early phases of differentiation, further supporting its role in adipocyte development [29]. Although the role of p38 in adipogenesis was contentious, studies supporting the involvement of phosphorylated p38 in adipogenesis have increased [29,30,31]. A recent study by Perumal et al. [31] showed the inhibitory effect of a p38 inhibitor, TAK-715, on the adipocyte differentiation of 3T3-L1 and human adipose stem cells (hASCs), mediated through deactivation of C/EBPα, PPARγ, signal transducer and activator of transcription 3 (STAT3), activating transcription factor 2 (ATF2), and p38 MAPK. Accordingly, the reduced phosphorylation of AKT, ERK, and p38 observed in NMA-treated cells suggests that modulation of these signaling pathways may be associated with the suppression of adipocyte differentiation and early-stage MCE.

To further elucidate the signaling dynamics, we analyzed AMPK activation, a key energy sensor that influences adipogenesis. The activation of AMPK inhibits the expression of C/EBP, PPAR, and SREBP1c, thereby hindering preadipocyte differentiation [32]. The significant decreases in p-AKT/AKT and the increases in p-AMPK/AMPK and p-ACC/ACC ratios were consistent, especially at 6 μM in the NMA-treated groups, suggesting inter-regulation between the PI3K-AKT and AMPK-ACC pathways, which occurs in an energy-deprived state [33]. Reduced GLUT4 expression and glucose uptake, together with a decrease in the p-AKT/AKT ratio, may be associated with alterations in cellular metabolic status in NMA-treated cells. These changes may contribute to AMPK activation. The phosphorylation of AMPK at Thr-172 results in the inhibitory phosphorylation of ACC, decreases the malonyl-CoA extender units for fatty acid synthesis, and increases catabolic processes, which favor ATP synthesis [32,34].

In addition, our data demonstrate that NMA is beneficial in suppressing adipogenesis, and that glucose uptake and the insulin signaling pathway may contribute to this effect. The potential metabolic implications of NMA for insulin resistance-like effects should be investigated further in in vitro models of other insulin-sensitive tissues, such as the liver and muscle, for a comprehensive understanding.

Furthermore, we determined the effect of NMA on lipid accumulation in human adipocytes and assessed its preliminary activity in human cells, supporting translational relevance. Similarly, NMA reduced lipid accumulation and glucose uptake in PCS-210-010 cells without detectable cytotoxicity. However, it should be noted that the effects of NMA were weaker in human PCS-210-010 cells compared with murine 3T3-L1 cells. Reductions in lipid accumulation and glucose uptake were observed at 3 and 6 μM during the differentiation stage, suggesting potential species-specific differences in responsiveness. Further studies are required to validate the molecular mechanisms in human systems.

To deepen understanding of NMA’s molecular targets, we conducted in silico studies focused on ERK1, whose BE value was the lowest among the protein targets, indicating the strongest binding affinity. ERK1 is a known regulator of adipogenesis and plays a crucial positive role in lipid accumulation during adipocyte differentiation, particularly through the interplay between p62 and ERK1, a key regulatory mechanism for adiposity [35]. ERK2 is also involved in adipogenesis, as inhibition of ERK1/2 suppresses this process; however, its specific role in lipid accumulation relative to ERK1 is less clear [30]. Based on the predicted BE compared with a reference compound, only ERK1 was selected for further all-atom MD simulations.

This structural insight was further supported by molecular dynamics simulations, reinforcing ERK1 as a potential therapeutic target. This computational method has been widely utilized in drug discovery and development, particularly for accelerated drug repurposing, hit identification, and hit-to-lead optimization [36,37]. The binding overlap with known ERK inhibitors supports the hypothesis that NMA may act through similar inhibitory mechanisms.

Many of the amino acid residues discussed here (Met55, Val56, Lys71, Leu173, Cys183, and Asp184) were also found in the binding of SCH772984, a selective and potent ERK1/2 inhibitor (PDB ID: 4QTB), visualized by BIOVIA Discovery Studio Visualizer 2021 [38]. This suggests that NMA may occupy the same site and exhibit a binding pattern closely similar to that of the previously identified inhibitor. Predicting these influential amino acids may provide clues for the further development of effective and selective compounds targeting ERK1.

As demonstrated above, NMA suppresses adipogenesis, especially during differentiation, by inhibiting the MCE process and the PI3K–AKT, ERK, and p38 MAPK pathways while activating the AMPK–ACC pathway. Similar inhibitory effects on early adipogenesis have been reported for ginsenoside CK [39]. Here, we report the anti-adipogenic activity of the acridone alkaloid, NMA. In addition, some limitations in this work should be noted. The findings are based on in vitro models, which may not fully reflect the complexity of in vivo metabolic regulation. Moreover, end-point measurements may not capture the temporal dynamics of adipogenic processes. Therefore, future investigations incorporating time-course analyses and appropriate animal models for anti-obesity would be valuable for further clarifying the mechanisms and evaluating the therapeutic potential of NMA.

## 4. Materials and Methods

### 4.1. Test Compounds

Prenylated acridone alkaloids [NMA, NMCA] from *Glycosmis parva* were isolated by Chansriniyom C. as described in a previous report [40]. NMR and HRESIMS spectra were re-examined and recorded on a Bruker Avance NEO 400 MHz instrument (Billerica, MA, USA) and an Agilent 6540 UHD Accurate-Mass Q-TOF spectrometer (Santa Clara, CA, USA), respectively (Figures S1–S3 and S5–S7, Tables S1 and S2). Their purity (>95%) was assessed using an HPLC Agilent 1290 Infinity instrument (Agilent Technologies, Santa Clara, CA, USA) at 254 nm (Figures S4 and S8). A stock concentration of 30 mM was prepared by dissolving the test compounds in dimethyl sulfoxide (DMSO; Sigma-Aldrich, St. Louis, MO, USA) and storing at −20 °C until use.

### 4.2. Chemicals, Biomolecules, Culture Media, and Assay Kits

Acetone (Cat No. #179124), chloroform (Cat No. #319988), dexamethasone (Cat No. #D9184), diethyl ether (Cat No. #346136), DMSO (Cat No. #D8418), isobutylmethylxanthine (IBMX, Cat No. #I5879), ORO (Cat No. #O0625), isopropanol (Cat No. #109634), methanol (Cat No. #319988), Triton X-100 (Cat No. #93443), bovine serum albumin (BSA, Cat No. #A9418), radioimmunoprecipitation assay (RIPA, Cat No. #R0278) buffer, and glucose (GO) assay kits (Cat No. #GAGO20), were purchased from Sigma-Aldrich (St. Louis, MO, USA). L-Glutamine (Cat No. #25030081), penicillin-streptomycin solution (10,000 U/mL, Cat No. #15140122), fetal bovine serum (FBS, Cat No. #10270106), trypsin (Cat No. #25200072), and Dulbecco’s Modified Eagle Medium (DMEM, Cat No. #12800017) were obtained from Gibco (Gaithersburg, MD, USA). 3-(4,5-Dimethylthiazol-2-yl)-2,5-diphenyltetrazolium bromide (MTT, Cat No. #M6494), enhanced chemiluminescence (ECL) assay (Cat No. #34580), and bicinchoninic acid (BCA, Cat No #23225) protein assay kits were purchased from Thermo Fisher (Rockford, IL, USA). A phosphatase Inhibitor Cocktail Tablets and Protease Inhibitor Cocktail Tablets (Cat No. #4906845001 and #693159001) were purchased from Roche Applied Science (Indianapolis, IN, USA). Insulin (Cat No. #CF034) was obtained from Himedia (Mumbai, India). The fibroblast basal medium (FBM, ATCC^®^, PCS-201-330) and fibroblast growth kit-low serum (ATCC^®^, PCS-201-041) were acquired from the American Type Culture Collection (ATCC) (Manassas, VA, USA). Primary antibodies for β-actin (Cat. No. #4970), GAPDH (Cat. No. #5174), ACC (Cat. No. #676), p-ACC (Ser79, Cat. No. #11818), AMPKα (Cat. No. #5831), p-AMPKα (Thr172, Cat. No. #2535), AKT (Cat. No. #4691), p-AKT (Ser473, Cat. No. #4060), p44/42 MAPK (Erk1/2) (Cat. No. #4695), Phospho-p44/42 MAPK (Erk1/2) (Cat. No. #4370), p38 (Cat. No. #8690), p-p38 (Thr180/Tyr182, Cat. No. #4511), cyclin D1 (Cat. No. #2978), cyclin D3 (Cat. No. #2936), CDK2 (Cat. No. #2546), CDK4 (Cat. No. #12790), CDK6 (Cat. No. #13331), p18^INK4C^ (Cat. No. #2896), p21^Waf1/Cip1^ (Cat. No. #2947), p27^Kip1^ (Cat. No. #3686), PPARγ (Cat. No. #2435), C/EBPα (Cat. No. #8178), FABP4 (Cat. No. #50699), adiponectin (Cat. No. #2789), FAS (Cat. No. #3180), PLIN1 (Cat. No. #9349), Anti-rabbit IgG, HRP-linked Antibody (Cat. No. #7074), Anti-mouse IgG, HRP-linked Antibody (Cat. No. #7076), GLUT1 (D3J3A) Rabbit mAb (Cat. No. #12939), and GLUT4 (1F8) Mouse mAb (Cat. No. #2213) were purchased from Cell Signaling Technology (Danvers, MA, USA). Primary antibodies specific to LPL (Cat. No. #PA5-85126) and SREBP1 (Cat. No. #PA1-337), and secondary antibodies, Alexa Fluor^TM^ 488 goat anti-rabbit IgG (H+L) (Cat. No. #A-11034) and Alexa Fluor^TM^ 488 goat anti-mouse IgG (H+L) (Cat. No. #A-11001), and Alexa Fluor™ 568 Phalloidin (Cat. No. #A-12380) were obtained from Invitrogen (Waltham, MA, USA).

### 4.3. Cell Culture, Differentiation, and Treatment

Mouse embryonic 3T3-L1 preadipocytes (CL-173 ^TM^, passages 5–15) and human subcutaneous preadipocytes (PCS-210-010^TM^, passages 3–6) were purchased from the ATCC. 3T3-L1 cells were cultured in DMEM containing 10% FBS, 1% penicillin-streptomycin solution, and 1% L-glutamine (200 mM) in a humidified 5% CO_2_ incubator at 37 °C, whereas PCS-210-010 cells were cultured with FBM and fibroblast growth kit-low serum.

For differentiation into mature adipocytes, preadipocytes (3T3-L1 and PCS-210-010) were grown as monolayers over 80% confluence for about 2–4 days and incubated in a differentiation medium consisting of FBM or DMEM supplemented with 10% FBS, 0.5 mM IBMX, 1 μM dexamethasone, and 5 μg/mL insulin for 2 days. The differentiation medium was replaced with a culture medium containing 5 μg/mL insulin, and the cells were incubated for an additional 2 days. The cells were maintained in the complete medium and refreshed every 2 days until cellular lipid droplets were observed.

Each test compound was dissolved in DMSO to obtain a 20 mM stock solution. The stock was further diluted in culture medium prior to treatment, with DMSO maintained at 0.5% (*v*/*v*) of the final volume in each well. Various concentrations of the test compounds were administered to 3T3-L1 cells at different stages of adipogenesis. To assess their effects during the differentiation stage, the compounds were added concurrently with the differentiation medium, and cells were harvested on day 4–5 of post-induction, corresponding to the onset of visible lipid accumulation during the intermediate differentiation stage. To evaluate their effects on mature adipocytes, the compounds were introduced on day 8 of the differentiation program, when substantial intracellular lipid accumulation was evident under microscopy, followed by an additional 2-day incubation prior to sample collection.

In addition, the experimental design and treatment schedule used in this study are shown in Table S3.

### 4.4. Cytotoxicity Assay

The MTT assay was used to assess cell viability. Briefly, cells were seeded in a 96-well plate at a density of 1 × 10^4^ cells/well and incubated at 37 °C with 5% CO_2_ until reaching 80% confluence. Differentiation was then initiated using differentiation media. The cells were then treated with various concentrations (1.5, 3, 6, 12.5, 25, 50, 100 μM, final concentrations) of the test compounds dissolved in DMSO, with the final DMSO concentration remaining below 0.5% (*v*/*v*) on Day 0 and Day 8 to assess their effects during the differentiation and maturation stages, respectively. After the incubation period described above, the medium was replaced with MTT solution (0.45 mg/mL in phosphate-buffered saline (PBS)) and incubated for 3 h in the dark. DMSO was added to dissolve the formazan crystals formed by the viable cells, and the absorbance was measured at 570 nm using a microplate reader (PerkinElmer, Waltham, MA, USA). Cells cultured with 0.5% DMSO served as a control group. Cell viability was calculated as a percentage of the control using the formula: % Cell viability = (A_treatment_/A_control_) × 100, where A_treatment_ and A_control_ represent the absorbances in the presence and absence of the test compounds, respectively. Non-cytotoxic concentrations were determined by the absence of a significant difference in cell viability relative to control cells.

### 4.5. Cell Proliferation and Cell Cycle Analyses

To assess the proliferation of 3T3-L1 cells in the presence of the test compounds, non-cytotoxic concentrations of the compounds were added for various periods at the early differentiation stage (24 and 48 h). The cells were grown as a monolayer in 96-well plates at a density of 5 × 10^3^ cells/well for 2 days, then exposed to a differentiation medium containing the test compounds. After incubation, the cells were fixed with 4% formaldehyde for 30 min and stained with 0.05% crystal violet solution for 30 min. Excess dye was removed by washing with deionized water, and the stained cells were air-dried overnight. Crystal violet was dissolved in methanol, and the absorbance was measured at 570 nm using a microplate reader. The percentage of cell proliferation was determined relative to the corresponding control groups cultured without differentiation medium at 24 h.

For cell cycle analysis, cells were seeded into 6-well plates as a monolayer in differentiation medium and then treated with or without the test compounds for 48 h. Cells were harvested by trypsinization and centrifuged at 2500× *g* for 5 min at 4 °C, and fixed in 70% cold ethanol at −20 °C overnight. The cells were washed with PBS (pH 7.4) and stained with 50 μg/mL propidium iodide (PI) containing 5 μg/mL RNase for 30 min at 37 °C) in the dark. The DNA content was analyzed by flow cytometry (Guava easyCyte, EMD Millipore, Austin, TX, USA) to determine the distribution of cells in the G0/G1, S, and G2/M phases of the cell cycle. Data were processed using InCyte 3.3 software and FlowJo V10 (Trial version, Williamson Way, Ashland, OR, USA) to calculate the percentage of cells in each phase.

### 4.6. Lipid Droplet Content Determination

ORO staining was performed to assess the lipid droplet content in differentiated and mature adipocytes. 3T3-L1 cells were seeded at 2 × 10^4^ cells/well in 24-well plates prior to adipogenic induction. After compound treatment as described above, the cells were washed with PBS and fixed with 10% formalin at room temperature (RT) for 45 min or longer. The fixed cells were rinsed twice with distilled water, briefly washed with 60% (*v*/*v*) isopropanol for 5 min at RT, and air-dried before staining with ORO working solution for 10 min at RT. Excess stain was removed by washing three times with distilled water, followed by microscopic image capture (Nikon Ts2, Tokyo, Japan). After complete removal of residual water and air-drying, ORO retained in lipid droplets was eluted with 100% isopropanol, and absorbance was measured at 510 nm. The ORO content was normalized to total protein levels determined by the BCA assay kit (Thermo Scientific, Rockford, IL, USA), following the manufacturer’s instructions and expressed as a percentage relative to the control group.

### 4.7. Glucose Uptake Assay

To determine glucose uptake activity, glucose levels in the media were measured using a GO assay kit after 48 h treatment with the compounds, based on the manufacturer’s instructions. Briefly, the collected media were diluted with deionized water at a 1:150 ratio, and 25 μL was mixed with 50 μL of the glucose-detecting reagent and incubated for 30 min. Next, 50 μL of 6 M sulfuric acid was added to generate a stable pink product corresponding to the glucose concentration. The absorbance was measured at 540 nm using a microplate reader, and the glucose remaining in the culture media was calculated.

### 4.8. Immunofluorescent Labeling Assay for GLUT Expression

To examine GLUT expression in 3T3-L1 adipocytes, cells were treated with the test compounds. After treatment, cells were washed three times with ice-cold PBS and fixed with 4% formalin for 10 min at RT. The cells were permeabilized with 0.1% Triton X-100 for 10 min at RT and blocked with 3% BSA. Primary antibodies against GLUT1 (D3J3A, rabbit monoclonal antibody; 1:200) or GLUT4 (1F8, mouse monoclonal antibody; 1:200) were applied, and the cells were incubated overnight at 4 °C. After washing with PBS, the cells were incubated with Alexa Fluor™ 488–conjugated secondary antibodies (goat anti-rabbit IgG for GLUT1 or goat anti-mouse IgG for GLUT4; 1:500) for 90 min at RT in the dark. F-actin was visualized by incubation with fluorescently labeled phalloidin (1× working solution) for 30 min at RT, followed by nuclear counterstaining with Hoechst 33342 (1:1000) for 10 min at RT in the dark. After final PBS washes, images were acquired using a confocal microscope (Zeiss LSM 900 with Airyscan 2, Jena, Germany).

### 4.9. Western Blot Analysis

Western blot analysis was performed to evaluate the effects of the test compounds on protein expression (3T3-L1 cells, 1 × 10^5^ cells/well in 6-well plates). After compound treatment as described above, cells were washed with ice-cold PBS and lysed in RIPA buffer supplemented with protease and phosphatase inhibitor cocktail tablets on ice for 30 min. The lysates were centrifuged at 13,000 rpm for 15 min at 4 °C, and the supernatants were collected. Protein concentrations were determined using a BCA protein assay. Equal amounts of protein (30 μg) were separated by 10% sodium dodecyl sulfate–polyacrylamide gel electrophoresis (SDS–PAGE) and transferred onto nitrocellulose membranes. The membranes were blocked with 5% BSA in Tris-buffered saline containing Tween 20 (TBST, (100 mM Tris-base, 150 mM NaCl, 0.1% Tween 20)) for 1 h at RT and incubated overnight at 4 °C with the appropriate primary antibodies (Table S4). After three times washing with TBST, the membranes were incubated with horseradish peroxidase–conjugated secondary antibodies (1:5000) for 1 h at room temperature. Protein bands were detected using an ECL reagent. Chemiluminescent signals were captured using X-ray film (Carestream, New York, NY, USA; n = 1, N1) or the iBright™ 1500 Imaging System (Thermo Fisher Scientific, Rockford, IL, USA; n = 2, N2 and N3). For film detection, membranes were incubated with ECL reagent and exposed to X-ray film in a darkroom for 0.5–30 min, depending on signal intensity, followed by standard development and fixation procedures. Digital detection was performed using the iBright™ 1500 Imaging System according to the manufacturer’s instructions. Images were analyzed using ImageJ version 1.53r, and band intensities were quantified and normalized to β-actin or GAPDH as internal loading controls.

Precision Plus Protein^TM^ Dual Color Standards (BIO-RAD Laboratories, Hercules, CA, USA, Cat. No. #1610374) were used as molecular weight markers for both detections. Biotinylated Protein Ladder (Cat. No. #7727) and Anti-biotin HRP-linked Antibody Cat. No. #7075), both from Cell Signaling Technology (Danvers, MA, USA), were used to visualize molecular weight markers on X-ray films.

### 4.10. Molecular Docking

The three-dimensional structures of four targets, including PKB (PDB ID: 1O6L) [41], human ERK1 (PDB ID: 4QTB) [38], ERK2 (PDB ID: 4QTA) [38], and p38α MAPK (PDB ID: 3ZSH) [42], were obtained from the RCSB protein data bank (https://www.rcsb.org/ (accessed on 17 December 2025)). The ligand NMA was manually sketched and subsequently optimized using Gaussian 16 at the B3LYP/6-31g(d) basis set [43]. The compound binding sites as x-y-z coordinates were determined based on the position of their respective cocrystallized compounds, with a uniform box size of 20 Å applied across all dimensions. The docking procedure was validated by redocking the cocrystallized compounds, followed by a visual inspection of the ligand conformation and orientation, as depicted in Figure S9. Upon validation, this docking protocol was subsequently used to evaluate the binding affinity of NMA, measured as the “binding energy (BE)” in kcal/mol. All docking calculations were performed on a Linux operating system using the Autodock VinaXB program [44]. In addition, binding energies (kcal/mol) of SCH772984 with ERK1 and ERK2, as predicted by molecular docking using AutoDock VinaXB are shown in Table S5.

### 4.11. MD Simulations

The initial complex structure was obtained from the docking pose predicted by the Autodock VinaXB program. The ionizable amino acid protonation states were assigned at physiological pH (pH = 7.4) using the PDB2PQR web interface [45]. The partial atomic charges of the compounds were approximated using the General AMBER force field version 2 (GAFF 2) available in the Antechamber module of AMBER 20. The complex was simulated under the periodic boundary condition using the AMBER ff19SB force field [46]. The isothermal-isobaric (NPT) scheme (310 K, 1 atm) and TIP3P water model were included. The topology and coordinates were gradually and structurally minimized using the SANDER and PMEMD modules of the AMBER20 software package. Electrostatic interactions were handled by particle mesh Ewald summation, and the SHAKE algorithm was applied to constrain all hydrogen atoms [47,48]. Temperature equilibration was achieved using the Langevin thermostat, whereas the pressure was controlled using the Berendsen barostat [49,50,51]. MD production was conducted for 500 ns. The post-dynamic trajectories were analyzed in terms of their structural features and relevant parameters using the CPPTRAJ module. Relative binding free energy and decomposition (${\Delta G}_{bind}^{residue}$) were performed using Molecular Mechanics with Generalized Born and Surface Area Solvation (MM/GBSA), neglecting the entropic term to reduce the computational cost and time.

### 4.12. Statistical Analysis

Statistical analyses were conducted using data from 3 independent experiments, and results were reported as means ± standard deviations (SD). A one-way analysis of variance was performed to compare the means, followed by a post hoc test where applicable, using GraphPad Prism version 9.3.0 software (GraphPad Software Inc., San Diego, CA, USA). A *p*-value < 0.05 was considered statistically significant for all analyses. In addition, the correlation coefficient was determined using Microsoft Excel (Microsoft 365, Redmond, WA, USA).

## Data Availability

The raw data supporting the conclusions of this article will be made available by the authors on request.

## Supplementary Materials

The following supporting information can be downloaded at: https://www.mdpi.com/article/10.3390/ijms27093914/s1.
