# Supplementary material for: Anti-Adipogenic Effects of N-Methylatalaphylline in 3T3-L1 Cells Through Modulation of Metabolic and Mitogenic Signaling Pathways
Source: Int J Mol Sci. 2026 Apr 28;27(9):3914. doi: 10.3390/ijms27093914 (PMC13164502; doi:10.3390/ijms27093914)
Supplement: Supplementary file 1 [file ijms-27-03914-s001.zip › ijms-4265763-supplementary.pdf]

Supplementary information

Anti-adipogenic effects of *N*-methylatalaphylline in 3T3-L1 cells through modulation of metabolic and mitogenic signaling pathways

Noppawan Woramongkolchai[1,2], Chatchai Chaotham[3,4], Utid Suriya[5], Hnin Ei Ei Khine[4], Pattara Poungcho[2], Chaiyaboot Ariyachet[6], Chia-Hung Yen[7] and Chaisak Chansriniyom[2,8,*]

[1]Pharmaceutical Sciences and Technology Program, Faculty of Pharmaceutical Sciences, Chulalongkorn University, Bangkok 10330, Thailand.

[2]Center of Excellence in Natural Products and Nanoparticles, Chulalongkorn University, Bangkok 10330, Thailand.

[3]Department of Biochemistry and Microbiology, Faculty of Pharmaceutical Sciences, Chulalongkorn University, Bangkok, 10330, Thailand.

[4]Center of Excellence in Preclinical Toxicity and Efficacy Assessment of Medicines and Chemicals, Chulalongkorn University, Bangkok, 10330, Thailand.

[5]Department of Biochemistry, Faculty of Science, Mahidol University, Bangkok, 10400, Thailand.

[6]Department of Biochemistry, Faculty of Medicine, Chulalongkorn University, Bangkok, 10330, Thailand.

[7]Graduate Institute of Natural Products, College of Pharmacy, Kaohsiung Medical University, Kaohsiung, 807, Taiwan.

[8]Department of Pharmacognosy and Pharmaceutical Botany, Faculty of Pharmaceutical Sciences, Chulalongkorn University, Bangkok, 10330, Thailand.

[*]Corresponding author. E-mail: chaisak.ch@chula.ac.th

Table of Contents

Table	Description	Page
**S1**	NMR Spectroscopic Data (400 MHz, acetone-d_6) of NMA	3
**S2**	NMR Spectroscopic Data (400 MHz, acetone-d_6) of NMCA	5
**S3**	The experimental design and treatment schedule used in this study	7
**S4**	Western blotting experiments: Antibodies and target proteins	7
**S5**	Binding energies (kcal/mol) of SCH772984 with ERK1 and ERK2, as predicted by molecular docking using AutoDock VinaXB.	8

Fig.	Description	Page
**S1**	HRESIMS spectrum of *N*-methylatalaphylline (NMA)	9
**S2**	^{1}H-NMR (400 MHz, acetone-d_6) Spectrum of NMA	9
**S3**	^{13}C-NMR (100 MHz, acetone-d_6) Spectrum of NMA	10
**S4**	HPLC chromatogram of NMA	10
**S5**	HRESIMS spectrum of *N*-methylcyclo-atalaphylline-A (NMCA)	11
**S6**	^{1}H-NMR (400 MHz, acetone-d_6) Spectrum of NMCA	11
**S7**	^{13}C-NMR (100 MHz, acetone-d_6) Spectrum of NMCA	12
**S8**	HPLC chromatogram of NMCA	12
**S9**	Molecular docking validation: Redocking of co-crystallized structure into the binding site	13

Table S1 NMR Spectroscopic Data (400 MHz, acetone-d_6) of NMA

position	NMA [a)]		*N*-methylatalaphylline [b)]	
	δH (ppm), *J* (Hz)	δC (ppm)	δH (ppm), *J* (Hz)	δC (ppm)
1/ OH-1	14.55 (1H, *s*)	160.6	14.43 (1H, *s*)	159.1
2	-	110.1	-	106.6
3/ OH-3		162.1	-	161.4
4	-	108.4	-	108.3
4a	-	149.8	-	148.4
5/ OH-5		149.4	9.32 (1H,*br s*)	148.9
6	7.27 (1H, *dd*, 8.0, 1.6)	120.4	7.16 (1H, *m*)	123.9
7	7.16 (1H, *t*, 8.0)	123.6	7.07 (1H, *t*, 8)	119.7
8	7.77 (1H, *dd*, 8.0, 1.6)	117.1	7.78 (1H, *br d*)	115.5
8a	-	125.9	-	124.4
9	-	183.5	-	182.1
9a	-	107.9	-	109.7
10a	-	139.1	-	138
N-CH3	3.67 (3H, *s*)	48.6	3.61 (3H, *s*)	47.8
1′	3.45 (2H, *d*, 7.2)	22.1	3.48 (2H, *m*)	21.5
2′	5.25 (1H, *m*)	123.3	5.28 (1H, *m*)	122.9
3′	-	132.3	-	130.7
4′	1.79 (3H, *s*)	18	1.72 (3H, *s*)**	18
5′	1.66 (3H, *s*)	25.9	1.77 (3H, *s*)**	25.6
1″	3.60 (2H, *d*, 6)	27.1	3.48 (2H,*m*)	26.3
2″	5.37 (1H, *m*)	124.3	5.36 (1H, *m*)	123
3″	-	133.4	-	131.2
4″	1.79 (3H, *s*)	18.1	1.82 (3H, *s*)**	18
5″	1.71 (3H, *s*)	25.8	1.82 (3H, *s*)**	25.6

a) ^{1}H-NMR (400 MHz, acetone-d_6); ^{13}C-NMR (100 MHz, acetone-d_6).

b) The ^{1}H-NMR (100 MHz, $CDCl_3$) data were reported by Wu[1]. The ^{13}C-NMR (DMSO-d_6) data were reported by Banerji[2].

(1) Wu, T. S.; Furukawa, H.; Kuoh, C. S. Acridone alkaloids. IV. Structures of four new acridone alkaloids from *Glycosmis citrifolia* (Willd.) Lindl. *Heterocycles* **1982**, *19*, 1047-1051.

(2) Banerji, J.; Ghoshal, N.; Sarkar, S.; Patra, A.; Abraham, K.; Shoolery, J. N. Studies on Rutaceae: Part I□Carbon-13 NMR studies on acridone bases & structure of an alkaloid from *Atalantia wightii*. *Indian J. Chem., Sect. B* **1981**, *20B*, 835-838.

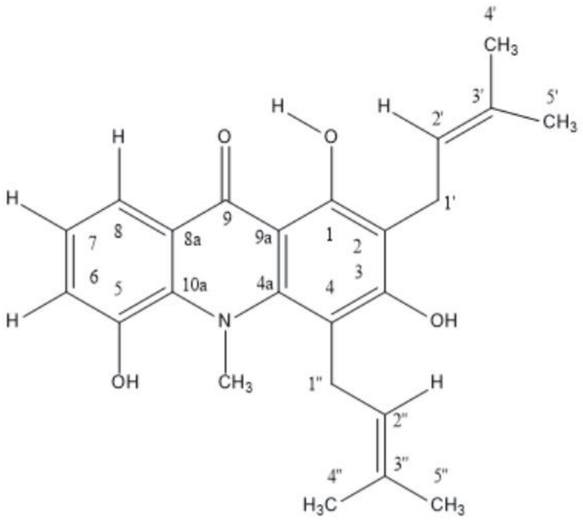

N-methylatalaphylline (NMA)

Table S2 NMR Spectroscopic Data (400 MHz, acetone-d_6) of NMCA

position	**NMCA** [a)]		*N*-methylcyclo-atalaphylline-A [b)]	
	δH (ppm), *J* (Hz)	δC (ppm)	δH (ppm), *J* (Hz)	δC (ppm)
1/ OH-1	14.66 (1H, *s*)	157.9	14.63 (1H, *s*)	157.5
2	-	104.3	-	103.4
3	-	159.7	-	158.8
4	-	109.4	-	108.5
4a	-	151.2	-	150
5/ OH-5	-	149.4	9.41 (1H, *br s*)	148.6
6	7.29 (1H, *dd*, 8.0, 1.6)	120.6	7.29 (1H, *br d*, 7.5)	119.7
7	7.18 (1H, *t*, 8.0)	124.0	7.18 (1H, *br d*, 7.5)	123.1
8	7.76 (1H, *dd*, 8.0, 1.6)	117.0	7.76 (1H, *d*, 7.5)	116.1
8a	-	125.7	-	124.9
9	-	183.6	-	182.7
9a	-	107.9	-	106.9
10a	-	138.9	-	138
N-CH3	3.72 (3H, *s*)	48.6	3.71 (3H, *s*)	47.7
1′	6.72 (1H, *d*, 10.0)	116.5	6.73 (1H, *d*, 9.9)	115.6
2′	5.70 (1H, *d*, 10.0)	127.9	5.70 (1H, *d*, 9.9)	126.9
3′	-	78.6	-	77.7
4′	1.48 (3H, *s*)	28.5	1.48 (3H, *s*)	27.6
5′	1.48 (3H, *s*)	28.5	1.48 (3H, *s*)	27.6
1″	3.51 (2H, *d*, 6.4)	26.6	3.51 (2H, *br d*, 6.3)	25.7
2″	5.36 (1H, *m*)	124.7	5.36 (1H, *m*)	123.9
3″	-	131.7	-	130.8
4″	1.70 (3H, *s*)	25.8	1.70 (3H,*s*)	24.9
5″	1.79 (3H, *s*)	18.2	1.80 (3H,*s*)	17.3

a) ^{1}H-NMR (400 MHz, acetone-d_6); ^{13}C-NMR (100 MHz, acetone-d_6).

b) The ^{1}H-NMR (300 MHz, acetone-d_6) and ^{13}C-NMR (75 MHz, acetone-d_6) data were reported by Chukaew[3].

(3) Chukaew, A.; Ponglimanont, C.; Karalai, C.; Tewtrakul, S. Potential anti-allergic acridone alkaloids from the roots of *Atalantia monophylla*. *Phytochemistry* **2008**, *69* (14), 2616-2620. DOI: 10.1016/j.phytochem.2008.08.007

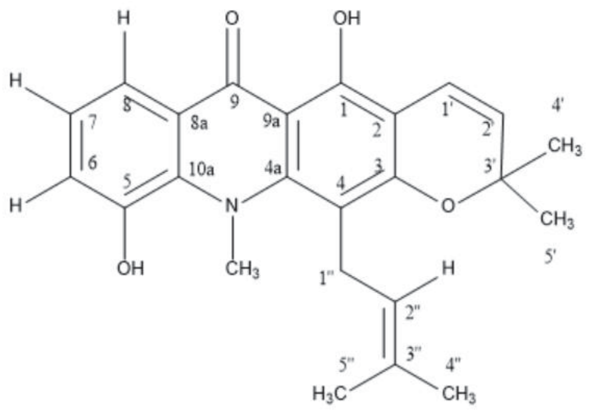

N-methylcyclo-atalaphylline-A (NMCA)

Table S3. The experimental design and treatment schedule used in this study

Stage	Timing	Experimental Design	Assays Performed
**Preadipocyte / Confluence**	Day −2 to 0	Cells cultured to reach ~80–100% confluence prior to induction	
**Early differentiation**	Day 0–2	Induction with adipogenic cocktail (MDI) and treatment with compounds during early differentiation stage	
Early differentiation	Day 2	Medium replaced with complete medium containing insulin	Cell viability (MTT), nuclear staining, cell cycle analysis, proliferation assay, MCE-mediated protein assay (Western Blot analysis)
**Differentiation (protein collection stage)**	Day 4–6	Cells treated with compounds during differentiation and harvested for molecular analysis	Western blot analysis (signaling pathways, adipogenic transcription factors, and downstream markers)
**Mature adipocyte (post-differentiation)**	Day 8–10	Fully differentiated adipocytes treated with compounds to assess metabolic function	Glucose uptake assay, Immunofluorescent staining (GLUT1/GLUT4), Oil Red O staining for the differentiation stage
**Late maturation**	Day 10-12	Endpoint analysis of mature adipocyte function	Cell viability (MTT), Nuclear staining, Glucose uptake assay, Immunofluorescence staining (GLUT1/GLUT4), Oil Red O staining, Western blot analysis

Table S4 Western blotting experiments: Antibodies and target proteins

Target protein	Host / Clone	Dilution (ratio)	Supplier	Cat. No.	Protein Loading (μg)
β-actin	Rabbit mAb	1:5,000	CST	#4970	30
GAPDH	Rabbit mAb	1:5,000	CST	#5174	30
ACC	Rabbit mAb	1:1,000	CST	#676	30
p-ACC (Ser79)	Rabbit mAb	1:1,000	CST	#11818	30
AMPKα	Rabbit mAb	1:1,000	CST	#5831	30
p-AMPKα (Thr172)	Rabbit mAb	1:1,000	CST	#2535	30
AKT	Rabbit mAb	1:1,000	CST	#4691	30
p-AKT (Ser473)	Rabbit mAb	1:1,000	CST	#4060	30

Target protein	Host / Clone	Dilution (ratio)	Supplier	Cat. No.	Protein Loading (μg)
Erk1/2 (p44/42 MAPK)	Rabbit mAb	1:1,000	CST	#4695	30
p-Erk1/2	Rabbit mAb	1:1,000	CST	#4370	30
p38 MAPK	Rabbit mAb	1:1,000	CST	#8690	30
p-p38 (Thr180/Tyr182)	Rabbit mAb	1:1,000	CST	#4511	30
Cyclin D1	Rabbit mAb	1:1,000	CST	#2978	30
Cyclin D3	Mouse mAb	1:2,000	CST	#2936	30
CDK2	Rabbit mAb	1:1,000	CST	#2546	30
CDK4	Rabbit mAb	1:1,000	CST	#12790	30
CDK6	Mouse mAb	1:1,000	CST	#13331	30
p18INK4C	Mouse mAb	1:1,000	CST	#2896	30
p21Waf1/Cip1	Rabbit mAb	1:1,000	CST	#2947	30
p27Kip1	Rabbit mAb	1:1,000	CST	#3686	30
PPARγ	Rabbit mAb	1:1,000	CST	#2435	30
C/EBPα	Rabbit mAb	1:1,000	CST	#8178	30
FABP4	Rabbit mAb	1:1,000	CST	#50699	30
Adiponectin	Rabbit mAb	1:1,000	CST	#2789	30
FAS	Rabbit mAb	1:1,000	CST	#3180	30
PLIN1	Rabbit mAb	1:1,000	CST	#9349	30
LPL	Rabbit pAb	1:1,000	Invitrogen	#PA5-85126	30
SREBP1	Rabbit pAb	1:1,000	Invitrogen	#PA1-337	30
GLUT1 (D3J3A)	Rabbit mAb	1:1000	CST	#12939	50
GLUT4 (1F8)	Mouse mAb	1:1000	CST	#2213	50

*CST: Cell Signaling Technology (Danvers, MA, USA)

Table S5. Binding energies (kcal/mol) of SCH772984 with ERK1 and ERK2, as predicted by molecular docking using AutoDock VinaXB

Targets	Binding Energy (kcal/mol)
**ERK1**	−14.0
**ERK2**	−10.4

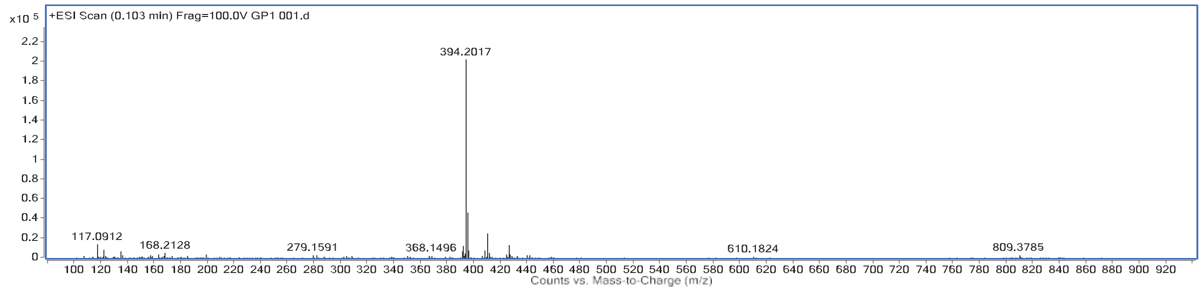

Fig. S1 HRESIMS spectrum of *N*-methylatalaphylline (NMA)

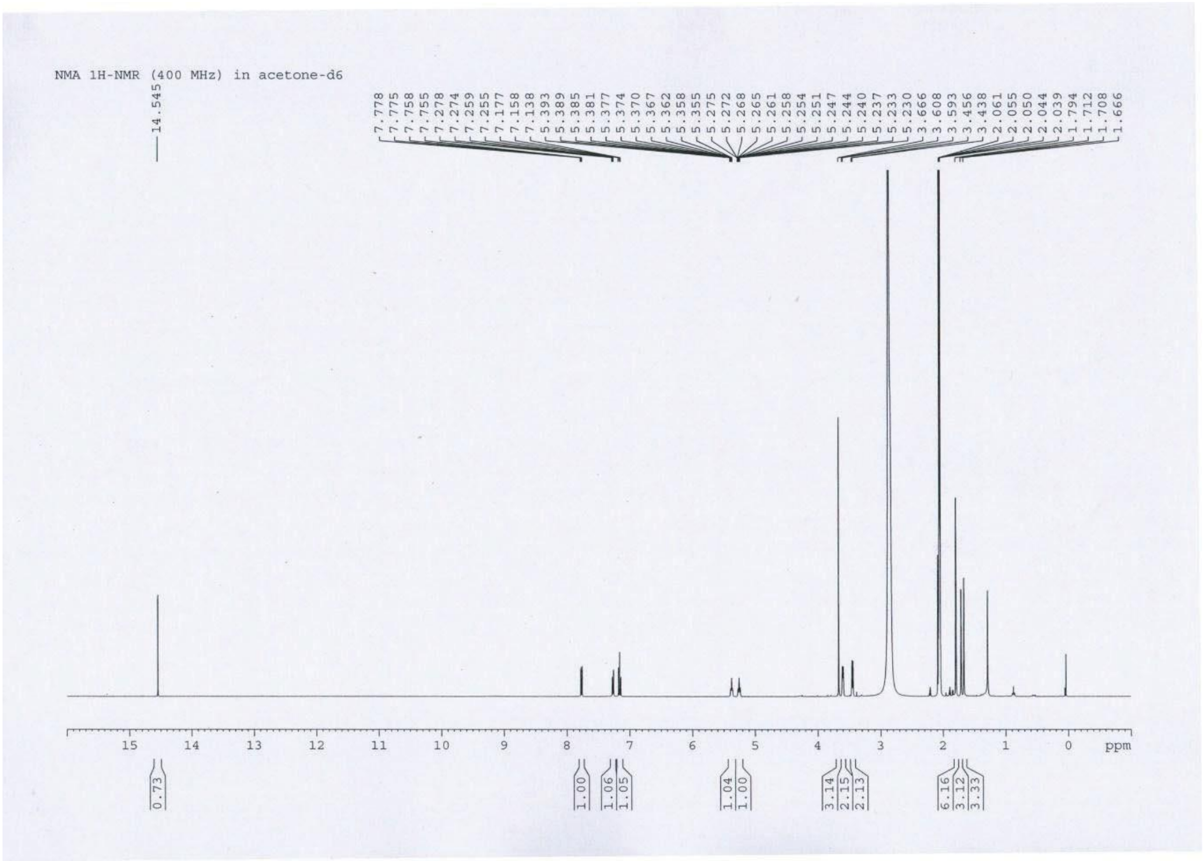

Fig. S2 ^{1}H-NMR (400 MHz, acetone-d_6) Spectrum of NMA

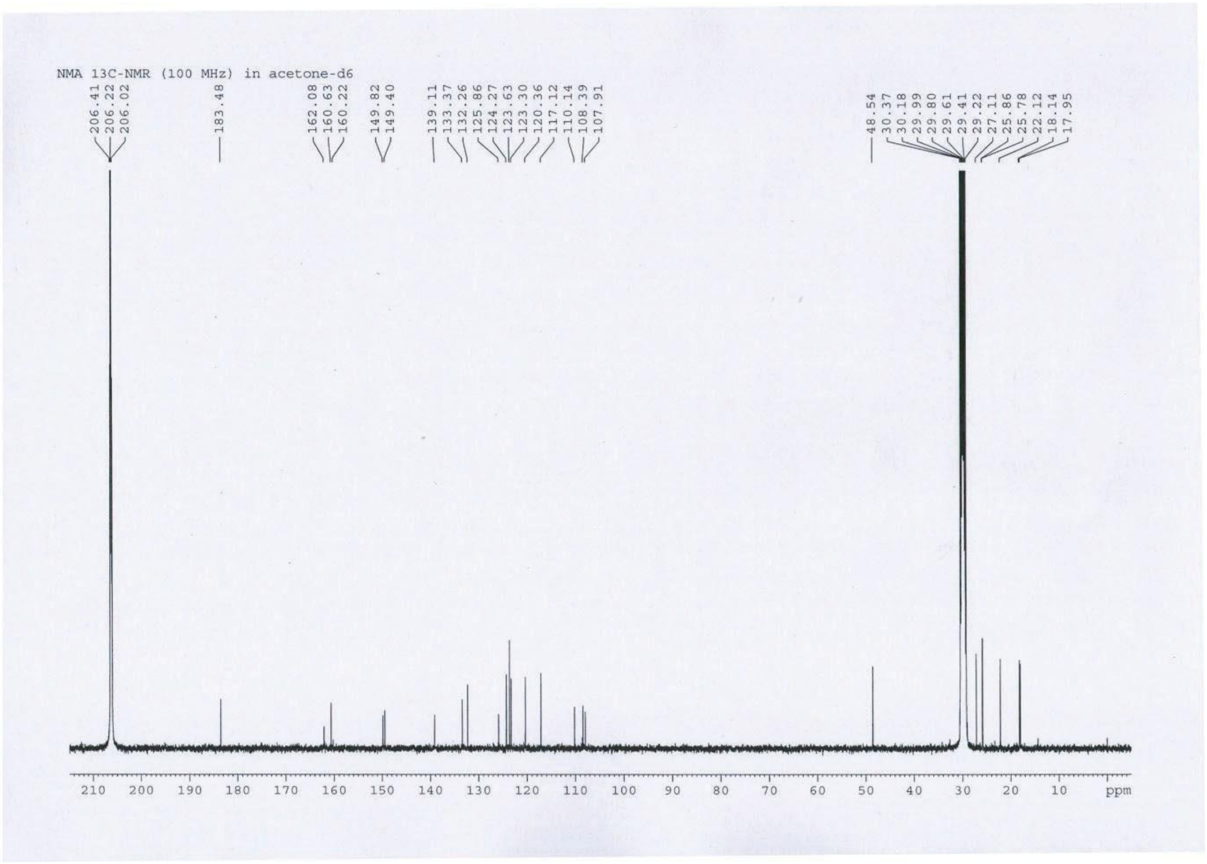

Fig. S3 ^{13}C-NMR (100 MHz, acetone-d_6) Spectrum of NMA

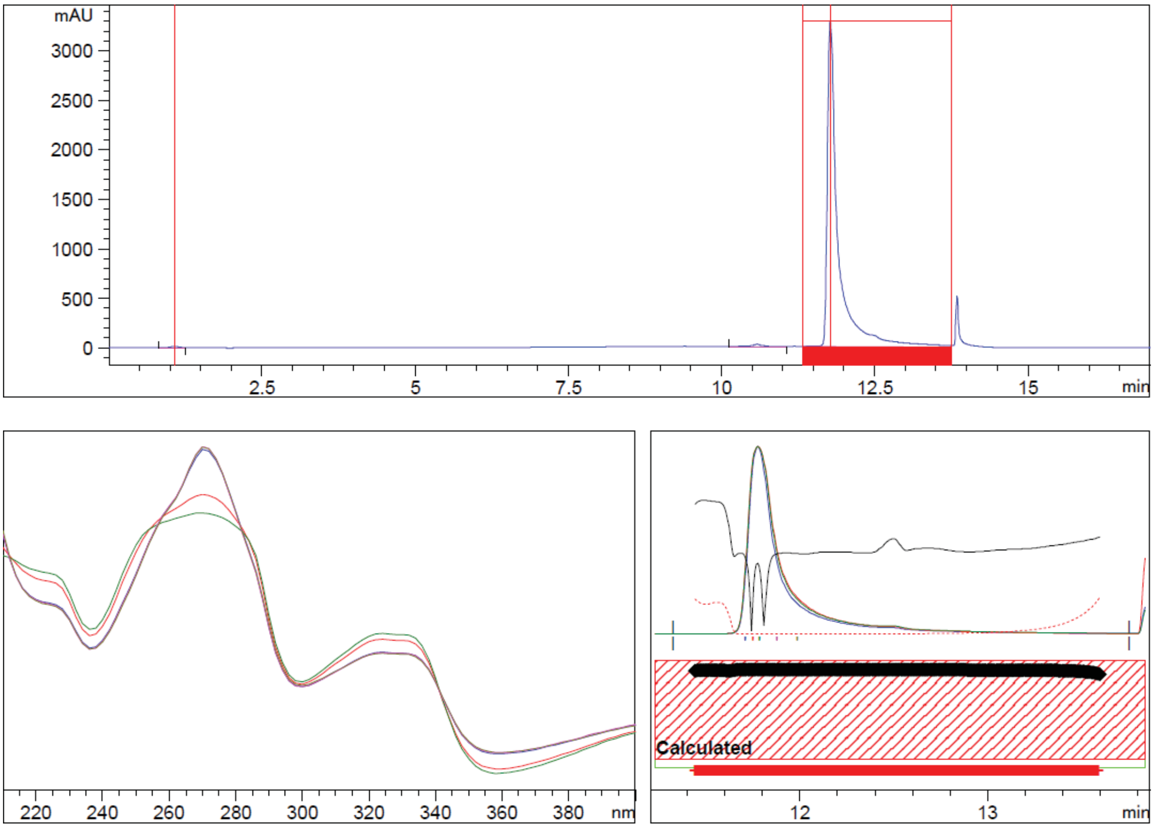

Fig. S4 HPLC chromatogram of NMA at t_R 11.781 min at 254 nm, (purity > 95%).

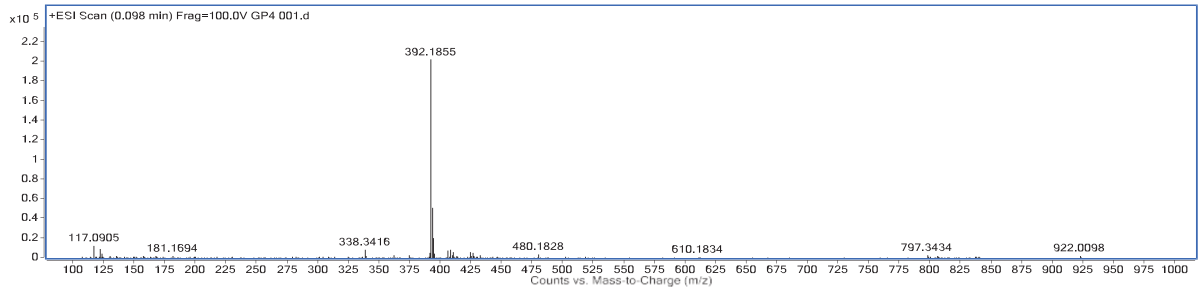

Fig. S5 HRESIMS spectrum of *N*-methylcyclo-atalaphylline-A (NMCA)

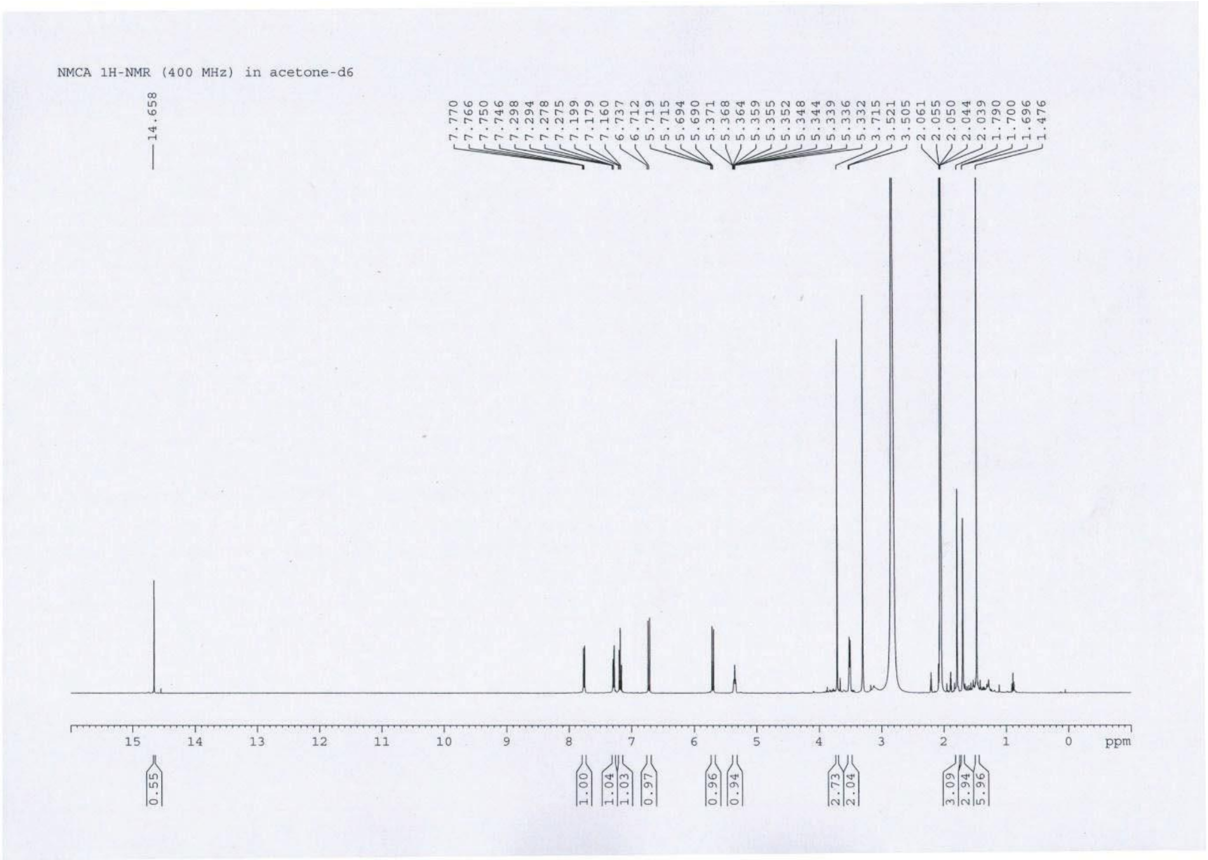

Fig. S6 ^{1}H-NMR (400 MHz, acetone-d_6) Spectrum of NMCA

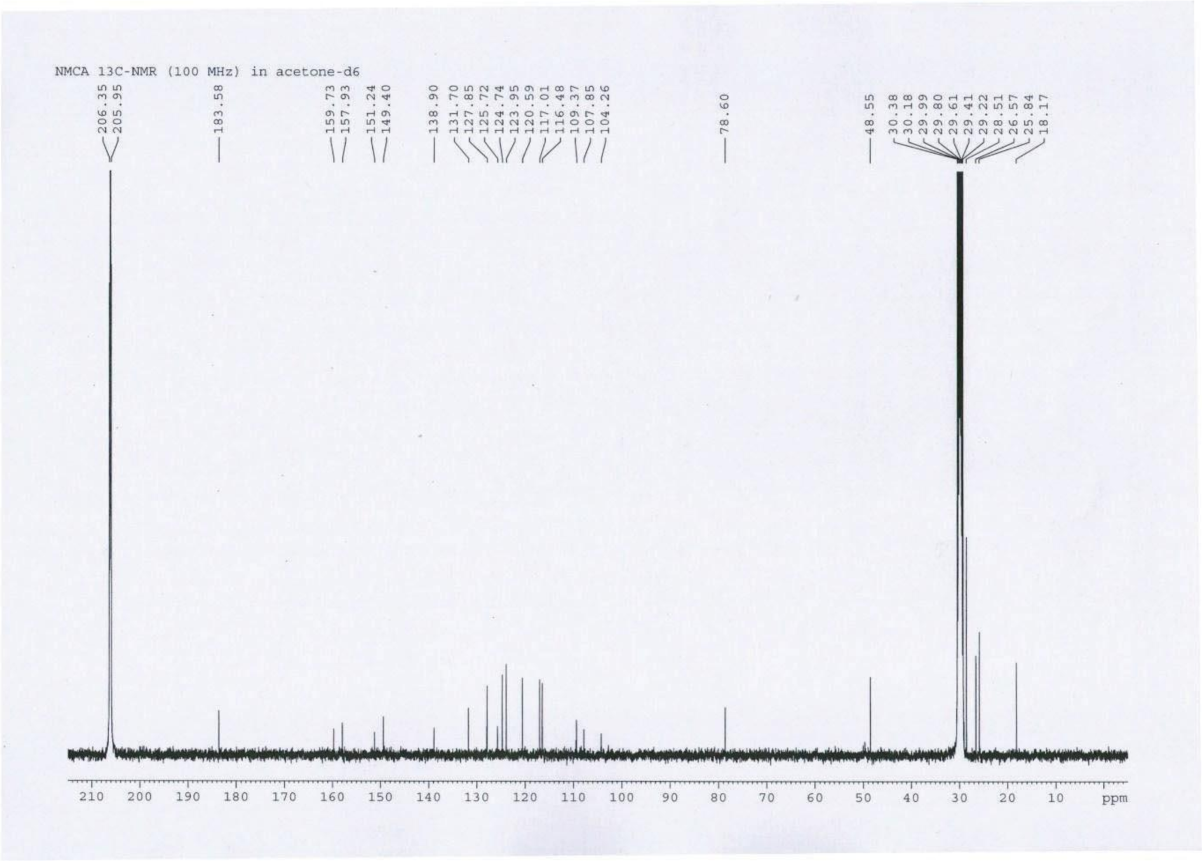

Fig. S7 ^{13}C-NMR (100 MHz, acetone-d_6) Spectrum of NMCA

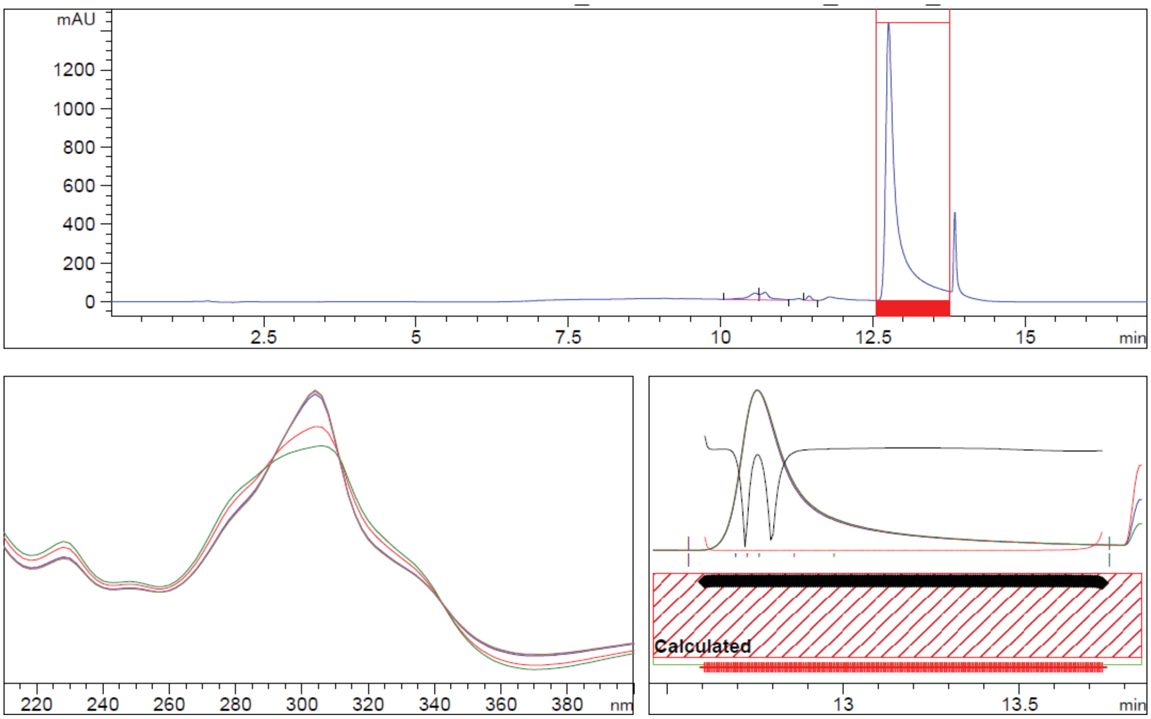

Fig. S8 HPLC chromatogram of NMA at t_R 12.762 min at 254 nm, (purity > 95%).

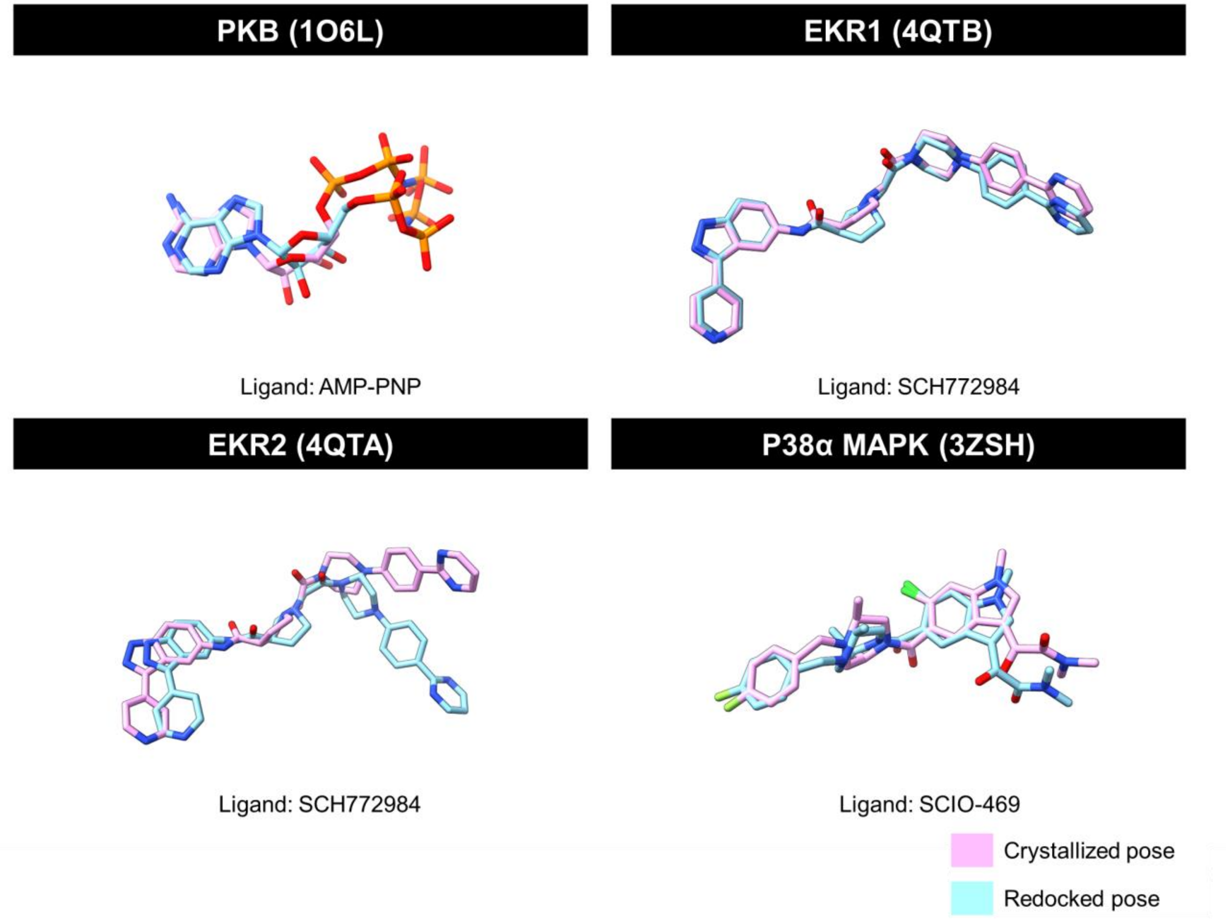

Fig. S9 Redocking of co-crystallized structure into the binding site, set in molecular docking simulation, and the alignment between redocked and original/crystallized poses, showing the parameters used in molecular docking could predict the ligand conformation closely similar to the experimental crystallized one (partly referred to as "validated docking protocol"). Note that the H atoms were not shown here.
